# Guidelines for laparoscopic treatment of ventral and incisional abdominal wall hernias (International Endohernia Society [IEHS])—Part III

**DOI:** 10.1007/s00464-013-3172-4

**Published:** 2013-09-17

**Authors:** R. Bittner, J. Bingener-Casey, U. Dietz, M. Fabian, G. Ferzli, R. Fortelny, F. Köckerling, J. Kukleta, K. LeBlanc, D. Lomanto, M. Misra, S. Morales-Conde, B. Ramshaw, W. Reinpold, S. Rim, M. Rohr, R. Schrittwieser, Th. Simon, M. Smietanski, B. Stechemesser, M. Timoney, P. Chowbey

**Affiliations:** 1 Hernia Center Rottenburg am Neckar, Winghofer Medicum, Röntgenstr.38, 72108 Rottenburg, Germany; 2 Division of Gastroenterological and General Surgery, Mayo Clinic, 200 First Street SW, Rochester, MN 55905 USA; 3 Department of General, Visceral, Vascular and Pediatric Surgery (Department of Surgery I), University Hospital of Wuerzburg, Oberduerrbacher Strasse 6, 97080 Wuerzburg, Germany; 4 Department of General Surgery, Halifax Health, Daytona Beach, FL USA; 5 Department of Surgery, Lutheran Medical Center, SUNY Health Science Center, Brooklyn, 65 Cromwell Avenue, Staten Island, NY USA; 6 Department of General, Visceral and Oncological Surgery, Wilhelminenspital, 1171 Vienna, Austria; 7 Department of Surgery and Center for Minimally Invasive Surgery, Vivantes Hospital, Neue Bergstr. 6, 13585 Berlin, Germany; 8 Department of General, Visceral, Abdominal Wall Surgery, Klinik Im Park, Grossmuensterplatz 9, 8001 Zürich, Switzerland; 9 Minimally Invasive Surgery Institute and the Fellowship Program, Surgeons Group of Baton Rouge of Our Lady of the Lake Physician Group, Baton Rouge, LA USA; 10 Minimally Invasive Surgical Center, KTP Advanced Surgical Training Center, YYL School of Medicine, National University Hospital, Kent Ridge Wing 2, 5 Lower Kent Ridge Road, Singapore, 119074 Singapore; 11 Division of Minimally Invasive Surgery, JPN Apex Trauma Centre, All India Institute of Medical Sciences, Angari Nagar, New Delhi, 110029 India; 12 Unit of Innovation in Minimally Invasive Surgery, University Hospital “Virgen del Rocío”, Sevilla, Spain; 13 Department of Surgery, Gross-Sand Hospital Hamburg, Gross-Sand 3, 21107 Hamburg, Germany; 14 Department of General Surgery, Katutura State Hospital, PO Box 81233, Olympia, Windhoek, Namibia; 15 Department of Surgery, LKH, Muerzzuschlag, Tragösserstrasse 1 und 1a, 8600 Bruck/Mur, Austria; 16 Department of Surgery, GRN-Klinik Sinsheim, Weinheim, Germany; 17 Ceynowa Hospital, Wejherowo, Poland; 18 Hernienzentrum Köln, Zeppelinstr.1, 50667 Köln, Germany; 19 Minimal Access, Metabolic, and Bariatric Surgery, Max Healthcare Institute Ltd., 2 Press Enclave Road, Saket, New Delhi India

## Section 7: Mesh technology

### Do we have an ideal mesh in terms of prevention of adhesions? Are coated meshes really necessary? Are there data to support the manufacturers’ claims of superiority? Is a permanent or absorbable barrier preferred?

#### F. Köckerling, D. Weyhe, M. C. Misra, U. Klinge, J. Kukleta

*Search terms:* “Incisional Hernia,” “Ventral Hernia,” “Laparoscopic Incisional Hernia Repair,” “Laparoscopic Ventral Hernia Repair,” “Hernia Repair and Meshes,” “Meshes,” “Mesh Repair,” “Laparoscopic Ventral Hernia Repair and Meshes,” “Incisional Hernia Repair and Meshes.”

A systematic search of the available literature was performed in July 2012 of Medline, PubMed, Cochrane Library, and relevant journals and reference lists using the above-listed search terms. The first search detected 78 relevant articles. In a second-level search, two articles were added. Twenty-six articles were thus used for this review.

##### Introduction

In general, clinical studies usually do not have sufficient power to confirm any claim of superiority of any device. Considering the heterogeneity of patients, surgeons, and procedures, a specific impact of the device to change the outcome is rarely possible with study cohorts of fewer than 1,000 patients per group. Thus, postmarket surveillance of devices is always supplemented by documentation in clinical registries. These will not be able to confirm any superiority, but they at least will help identify devices with poor performance.

Adhesions after laparoscopic ventral hernia repair (LVHR) is a common phenomenon, the result of the trauma of surgery and a reaction to the mesh and/or fixation devices. No technique or device completely prevents the formation of adhesions.

Direct contact of visceral organs with polypropylene (PP) and polyester is followed by dense adhesions to the mesh, leading to significant risk of bowel injury requiring resection during revision operations and suspected to be followed by a higher risk for development of an intestinal fistula. This risk is decreased with use of films (expanded polytetrafluoroethylene [ePTFE]) or textile meshes made of polyvinyl difluoride (PVDF), PP, or polyester, but with an additional coating/barrier function of another material, such as titanium, collagen, cellulose, hyaluronic acid, or polydioxanon.

Any film barrier covering a textile will initiate a tissue response comparable to that of the pure filmlike device with encapsulation of the entire prosthesis. Because any damage to peritoneum heals within days, a temporary protection of the polymer surface should be sufficient. However, whether this provides a sufficient protection depends to the textile material, and some require a permanent barrier.

##### Statements

**Table:** 

Level 4	Laparoscopic ventral and incisional hernia repair can be performed with the use of ePTFE, PVDF, or composite meshes and is appropriate for use within the abdominal cavity
Level 5	The results of experimental studies on large animals with LVHR and comparison of meshes show advantages of lightweight PP meshes vs. heavy-weight meshes, ePTFE and composite meshes vs. pure PP meshes, composite meshes vs. ePTFE meshes, and composite meshes vs. composite meshes
	After laparoscopic incisional hernia repair, adhesions will develop in at least two-thirds of the patients. Adhesions cannot be completely prevented by any of the materials used as intraperitoneal onlay meshes (IPOM), and consequently adhesions must expected in most patients
	Materials for use within the abdominal cavity can be made of ePTFE, PVDF, polyester, or PP; the latter needs an additional barrier to prevent any direct contact with the intestine (composite meshes). Unprotected porous PP and polyester meshes, which are placed in direct contact to the bowel, induce a higher risk for bowel erosion and/or bowel resection at subsequent surgery
	A low recurrence rate can be achieved if adequate technique is applied with all available materials
	Filmlike materials tend to show encapsulation and sometimes extensive shrinkage and require a method of permanent fixation
	Enterocutaneous fistulas after LVHR are rare events, particularly with ePTFE
	Experimental studies in animals showed contradictory results and are not strictly comparable
	Tissue integration of the various devices with different design characteristics differ and require different fixation techniques
	There is no ideal mesh, but every mesh has to be considered as a compromise with regard to strength, elasticity, tissue ingrowth, and cellular response, with its specific advantages and disadvantages
	Most devices demonstrate a lack of stretchability, so that folding or wrinkling of the fixed mesh after release of the pneumoperitoneum may be unavoidable

##### Recommendations

**Table:** 

Grade C	For laparoscopic incisional and ventral hernia repair, only materials approved for use in the abdominal cavity (PTFE, PVDF, and composite meshes) should be used. Meshes lacking approval for use within the abdominal cavity should not be used outside approved research
	It is difficult to eradicate bacteria from ePTFE, and therefore it should be removed (explanted) in the presence of severe contamination
Grade D	The final choice of mesh at the present time should be based on the surgeon’s preference while awaiting further data from controlled clinical trials
	Based on today’s knowledge, plain PP (without a protective layer) cannot be recommended for intra-abdominal use
	Fixation has to consider the specific flexibility and tissue integration of the device
	Quality control of outcome requires a long follow-up and should use registries with standardized sets of variables with an open-ended option for surveillance

When meshes are inserted intraperitoneally during laparoscopic intraperitoneal onlay meshes (IPOM), they must meet stringent requirements because they directly contact the intestines. Eriksen et al. [1] formulated the following characteristics for an optimal mesh to be used for laparoscopic repair of ventral and incisional hernias:

- Minimal adhesion formation.
- Excellent tissue ingrowth.
- Minimal shrinkage.
- No infection or fistula formation.
- Minimal pain.
- Minimal seroma formation.
- No change in abdominal wall compliance.
- Low price.
- Easy to manipulate.

Typically, meshes are made of the basic materials PP, polyester, polyvinylidenfluoride, or PTFE. The use of pure PP meshes and polyester meshes are not recommended for laparoscopic IPOM [1–3]. It is accepted that PP and polyester meshes are coated either with a protective membrane or a protective film (absorbable or nonabsorbable) or with a titanium layer to protect the viscera. These composite meshes, as they are known, and ePTFE meshes are generally recommended for intraperitoneal use [1, 2, 4, 5] (Table 1). It is assumed that the use of these meshes reduced few adhesion formation and hence lowered the risk of intestinal damage and fistula formation.

**Table 1:** Meshes approved for use in the abdominal cavity

Group	Name of mesh	Material	Company name
PTFE	Mycromesh	ePTFE	W. L. Gore
	DualMesh	ePTFE	W. L. Gore
	Dulex	ePTFE	C. R. Bard
	MotifMESH	cPTFE	Proxy Biomedical
	Omyramesh	cPTFE	Aesculap AG
PVDF	Dynamesh	PP/polyvinylidene fluoride	FEG Textiltechnik/Dahlhausen
Composite mesh with absorbable barrier coated	Glucamesh	PP with beta glucan coating	Genzyme
	Proceed	PP with ORC layer	Ethicon
	Sepramesh	PP with resorbable layer	Genzyme
	Parietene Composite	PP with collagen coating	Sofradim
	Parietex Composite	Polyester with collagen coating	Sofradim
	Physiomesh	PP with poliglecaprone 25	Ethicon
	C-Qur	PP with omega 3 fatty acid coating	Atrium Medical Corp.
	Ventrio ST	PP with PGA fibers and PDO filaments and hydrogel barrier	C. R. Bard
Composite mesh with permanent barrier coated	TiMesh	PP with titanium coating	pfm medical AG
	Composix	PP/ePTFE	C. R. Bard
	Ventrio Hernia Patch	PP/ePTFE	C. R. Bard
	Intramesh T1	PP/ePTFE	Cousin Biotech
	Intramesh W3	Polyester mesh with silicone layer	Cousin Biotech

*PTFE* polytetrafluoroethylene, *ePTFE* expanded PTFE, *cPTFE* condensed PTFE, *PVDF* polyvinyl difluoride, *PP* polypropylene, *ORC* oxidized regenerated cellulose

Modified after Eriksen et al. [1]

##### Clinical studies

To date, there has been a paucity of clinical case series and only one randomized trial providing general recommendations for specific meshes. Only a few clinically important differences that could be deemed to be clinically relevant outcome parameters have been discerned in comparative studies between the meshes.

In a prospective randomized trial, Moreno-Egea et al. [6] compared in laparoscopic incisional hernia repair the use of a lightweight titanium-coated mesh (*n* = 51) with a collagen–polyester composite mesh (*n* = 51). The primary end points were pain and recurrence. The secondary end points were morbidity and patient outcomes (analgesic consumption, return to everyday activities). The postoperative complication rates were similar for the two meshes. Pain was significantly less common in the titanium-coated mesh group at 1 month (*p* = 0.029). There was a significant difference between the two groups in the average use of analgesics in favor of the titanium-coated mesh group (*p* < 0.001). The titanium-coated mesh group returned to everyday activities after 6.9 days versus 9.7 days for the collagen–polyester composite mesh group (*p* < 0.001). The rate of recurrence did not differ between the two groups at the 2-year follow-up evaluation. The authors concluded that the light titanium-covered PP mesh was associated with less postoperative pain in the short term, lower analgesic consumption, and a quicker return to everyday activities than the Parietex composite medium-weight mesh.

In a retrospective comparative study, Colon et al. compared 116 patients who had undergone LVHR, 66 of whom received a polyester-based composite mesh and 50 a PTFE mesh [7]. No significant differences were noted in terms of recurrence rate, wound complications, mesh-related infections, or persistent pain with an average postoperative follow-up of 12 months. Chelala et al. [8] reported on the intraoperative findings of 85 reoperations after laparoscopic repair of ventral and incisional hernias with the polyester-based mesh Parietex Composite. They detected, after an average of 52 months, no adhesions in 47 % of cases, few adhesions in 42 %, and serosal adhesions in 11 %.

Jenkins et al. [9] presented 69 patients who underwent laparoscopic surgery after prior intraperitoneal mesh placement for ventral hernia repair. Previous meshes were absorbable-barrier-coated mesh in 18 cases (Proceed, Sepramesh, C-Qur, Parietex Composite), permanent-barrier composite meshes in 17 cases (Composix), permanent-barrier noncomposite mesh in 14 cases (DualMesh), uncoated PP mesh in 12 cases, and biologic mesh in 8 cases. Indications for laparoscopic reexploration were recurrent ventral hernia (*n* = 58), chronic pain (*n* = 3), cholecystectomy (*n* = 3), parastomal hernia (*n* = 2), small bowel obstruction (*n* = 1), nephrectomy (*n* = 1), and Nissen fundoplication (*n* = 1). Adhesions to DualMesh were less tenacious (*p* < 0.05) compared to all other meshes. Surface area of adhesions to DualMesh was less (*p* < 0.05) than Composix and uncoated PP mesh, but not absorbable-barrier-coated and biologic mesh. For adhesiolysis time, the mesh surface area was less (*p* < 0.05) for DualMesh compared to Composix, uncoated PP, and biologic mesh, but not to absorbable-barrier-coated mesh. Adhesiolysis-related complications occurred in two (16.7 %) (*p* = NS) patients with uncoated PP mesh, one cystotomy and one enterotomy; both were repaired laparoscopically. There were two (16.7 %) (*p* = NS) conversions to an open procedure: one converted patient had Composix (6.7 %) and one had absorbable-barrier-coated mesh (5.9 %). There were no adhesiolysis-related complications with these meshes. There were no adhesiolysis-related complications or conversions to open in the DualMesh or biologic mesh groups.

Wassenaar et al. [10] presented a series of 65 patients who had a subsequent abdominal operation after more than 1 month after a laparoscopic ventral and incisional hernia repair (65 of 695; 9.4 %) with DualMesh. Only one patient required acute surgical intervention, which was due to a laparoscopic ventral and incisional hernia repair-related adhesion (0.15 %). Laparoscopy was performed in 83 % and laparotomy in 17 % of the patients. Adhesions to the implant were present in 83 % of patients; in 65 % the adhesions involved omentum only, and in 18 % the bowel was involved. The required adhesiolysis was uncomplicated, and there were no inadvertent enterotomies.

Heniford et al. [11] reported on a consecutive series of 850 cases of laparoscopic IPOM for ventral and incisional hernias with ePTFE (DualMesh). They identified a complication rate of 13.2 %. Ileus was seen in 3.0 % and long-term seroma in 2.6 %. A recurrence was noted in 4.7 % with an average follow-up of 20 months. Koehler et al. [12] reported on 65 reoperations after laparoscopic IPOM with ePTFE (DualMesh). No adhesions were seen in 23 %, avascular adhesions in 68 %, and dense adhesions in 9 %.

Berger and Bientzle [13] reported on their experiences with 297 laparoscopic repairs of incisional hernias with PP/polyvinylidene fluoride (DynaMesh). In that series, mesh-related infections occurred in 1 % but did not result in removal of the mesh. The rate of intestinal fistulas was 0.34 %. A recurrence rate of 0.6 % was found, but no long-term mesh-related complications were noted. As opposed to the good experiences reported by Berger and Bientzle [13] with DynaMesh, Fortelny et al. [14] reported a higher complication rate after laparoscopic IPOM repair of incisional hernias with DynaMesh. After a follow-up examination period of 1 year, adhesions necessitating reintervention occurred in 5 of 29 patients, and in 3 of 29 cases the mesh had to be explanted (an infection in one case required excision). At present, the above reports represent the only large clinical case series with use of defined ePTFE, PVDF, or composite meshes. There are a few scattered reports that pure PP mesh has been used without serious complications.

In 2000, Chowbey et al. [15] reported on 202 LVHRs with the use of pure PP meshes without a barrier material (the product was not named). In their series, there were two postoperative hernia recurrences at a mean follow-up of 2.9 years. The incidence of seroma formation postoperatively was 32 % in the first 3 years but declined to 18 % subsequently with postoperative abdominal wall pressure dressings. There were no postoperative sequelae related to bowel adhesions. Halm et al. reported on 39 patients who underwent subsequent laparotomy/laparoscopy after prosthetic incisional hernia repair with intraperitoneally placed PP meshes [3]. The perioperative course was complicated in 76 % of procedures. Small bowel resections were necessary in 21 % of the cases. Twenty-six percent of the patients developed a surgical site infection. The authors concluded that the intraperitoneal positioning of PP mesh for incisional and ventral hernia repair should be avoided. Though not published, 60–70 % of laparoscopic ventral and incisional hernia repairs are undertaken with pure PP meshes in India because of the cost and affordability issues (personal communication of Misra 2012) [16].

##### Experimental studies

By means of several animal experimental studies, attempts were made to identify differences between the meshes. To that effect, investigations were conducted on both small animals and large animals. According to Penttinen and Grönroos [17], the closest models to surgical practice are those using large animals (swine or sheep), which allow the creation of hernias that resemble the human anatomy. On the basis of Eriksen et al. [1], only a few experimental studies have been performed in large animals with proper mesh size and the laparoscopic technique. Conze et al. [17] performed a study comparing heavy-weight (90 g/m^2^; pore size: 0.6 mm), medium-weight (45 g/m^2^, pore size: 2.5 mm), and lightweight (29 g/m^2^, pore size: 4 mm) pure PP meshes in LVHR. The heavy-weight, small porous PP mesh showed significantly stronger adhesion formation. Granuloma formation was lowest in large-pore monofilament meshes.

Borrazzo et al. [19] compared pure PP mesh, ePTFE (DualMesh), and PP coated on one side with a bioabsorbable adhesion barrier (Sepramesh). The mean area of adhesion formation was 14 % in the Sepramesh group, 40 % in the pure PP group, and 41 % in the ePTFE group. The difference between Sepramesh and pure PP was significant (*p* = 0.013).

Another study by Jacob et al. [20] compared a pure PP mesh with a mesh made of a polyester parietal layer and an antiadhesive collagen visceral layer (Parietex Composite) with a PP soft mesh encapsulated in a polydioxanone polymer film covered by a layer of absorbable oxidized regenerated cellulose (Proceed). The mean area of adhesion to Parietex Composite (11 %) was significantly less than for Proceed (48 %; *p* < 0.008) or pure PP (46 %; *p* < 0.008). Adhesion peel strength was significantly less for Parietex Composite (5.9N) than for Proceed (12.1N; *p* < 0.02) or pure PP (12.9; *p* < 0.02).

Comparison of the composite mesh TiMesh with titanium coating of the lightweight PP and ePTFE (DualMesh) showed a significantly higher shrinkage rate for ePTFE (*p* = 0.006) [21]. Determination of the partial volume of the inflammatory cells showed significantly lower median figures for TiMesh (*p* = 0.009). Measurements of the proliferation marker Ki-67 showed significantly higher volumes for ePTFE (*p* = 0.011). The apoptosis index was significantly higher for the ePTFE mesh (*p* < 0.002) [21]. Comparison of collagen-coated polyester (Parietex Composite) and Composite ePTFE/PP mesh (Composix) indicates that collagen-coated polyester (Parietex Composite) induces fewer adhesions (14.5 vs. 53.4 %; *p* = 0.007) [21].

Comparison of the two composite meshes Parietex Composite and DynaMesh showed a significant reduction of intra-abdominal adhesion formation for Parietex Composite [23].

Another comparison of a PP mesh with collagen coating (Parietene Composite) with a PP mesh with polyvinylidene fluoride on the visceral side (DynaMesh) and a PP mesh with polydioxanone and cellulose coating exhibited a markedly lower value of 12.8 % for Parietene Composite regarding adhesions to the greater omentum, and 31.7 % for Proceed and 33.2 % for DynaMesh (*p* = 0.01) [24]. A similar value of 14 % was obtained for shrinkage of DynaMesh and Parietene Composite, while Proceed showed a 25 % reduction in surface area (*p* = 0.029 vs. DynaMesh and *p* = 0.041 vs. Parietene Composite) [24]. Deeken et al. [25] compared the novel absorbable-barrier-coated mesh VentrioST with other absorbable-barrier meshes (Sepramesh and Proceed) and a permanent barrier mesh. A significantly greater area of percentage contraction was demonstrated for Proceed (26.9 %) compared to Ventrio (14.5 %), VentrioST (8.8 %), and Sepramesh (9.2 %). VentrioST demonstrated similar amounts of adhesion area, tenacity, and tissue ingrowth compared to the other meshes [25].

### Role of biological meshes in laparoscopic incisional and ventral hernia repair? Are they advantageous in infected abdominal wall?

#### B. Stechemesser, D. Weyhe, B. Ramshaw, F. Köckerling, G. S. Ferzli

*Search terms:* “Incisional Hernia,” “Ventral Hernia,” “Laparoscopic Incisional Hernia Repair,” “Laparoscopic Ventral Hernia Repair,” “Biological Meshes,” “Meshes and Hernia Repair,” “Biological Meshes and Hernia Repair.”

A systematic search of the available literature was performed in July 2012 of Medline, PubMed, Cochrane Library, and relevant journals and reference lists using the above-listed search terms. The first search detected 45 relevant articles. In a second-level search, one article was added. In summary, seven articles and studies were used for this review.

##### Statements

**Table:** 

Level 1b	The use of non-cross-linked biological meshes for elective laparoscopic bridging repair of incisional and ventral hernias shows a high recurrence rate
Level 3	Recurrence rate in elective laparoscopic repair of incisional and ventral hernias using a cross-linked acellular porcine dermal collagen implant is not significantly higher compared to synthetic composite mesh
Level 4	Biological meshes are not impervious to infection
	Laparoscopic repair of incisional and ventral hernias in an infected or potentially contaminated surgical field can be performed with non-cross-linked biological meshes but the defect should be closed with suture(s)

##### Recommendations

**Table:** 

Grade A	Elective laparoscopic repair of incisional and ventral hernias should not be performed with the use of non-cross-linked biological mesh with a bridging technique
Grade D	Caution is advised in the use of biological meshes in a contaminated field
	Laparoscopic repair of incisional and ventral hernias with non-cross-linked biological meshes in an infected or potentially contaminated surgical field may be a viable option if the hernia defect is closed primarily
	Elective laparoscopic repair of incisional and ventral hernias with cross-linked biological meshes can be considered a reasonable surgical option

In a systematic review of the implants available for treatment of incisional and ventral hernias by Shankaran et al. [2], biological meshes are listed as a possible alternative. In this respect, biological meshes can be used in an extraperitoneal as well as an intraperitoneal position. The main advantage cited for biological meshes is their suitability for use in contaminated and infected surgical fields. Because biological meshes are revascularized and incorporated into the host tissue, they provoke a markedly less pronounced foreign body reaction compared to synthetic meshes. The relatively low concentration of inflammatory cells around a biological mesh may explain their successful use in a contaminated field. According to Shankaran et al., numerous studies have demonstrated that biological meshes can be used in contaminated fields. However, a study of the publications included in the clinical review by Shankaran et al. reveals that only six publications were actually truly evaluated for that review. All publications were retrospective case series. Only two publications explicitly focused on usage in a contaminated setting. The number of cases varied between 9 and 75. Overall, the patient cohort is so heterogeneous that extreme caution is advised when assessing the statement made by Shankaran et al. on the use of biological meshes in a contaminated situation.

Another systematic review from Bellows et al. [26] shows

that a paucity of high quality evidence exists in the peer-reviewed medical literature on the use of biological tissue grafts for incisional hernia repair. Although the rationale for using biological prosthesis for complex and contaminated incisional hernias is related to surgeons’ concerns regarding the potential dire consequences of using permanent mesh in contaminated fields, there are yet any published prospective clinical trials justifying their preference over conventional mesh materials. Until such evidence is forthcoming, the use of biological prosthetics in complex incisional hernia repairs should proceed with caution. There may very well be a solid place for the use of these materials, but for them to add true value to complex hernia repair, better-designed and reported studies are necessary to help guide clinical practice.

Although most xenografts are used by surgeons in the setting of contamination, none of these biological meshes has received a US Food and Drug Administration (FDA) indication for use in this situation [27]. One particular interesting study reviewed the FDA database of adverse events associated with biological mesh. One hundred fifty adverse events were identified, with 80 % described as infection and 90 % necessitating reoperation [27, 28].

##### Elective laparoscopic repair of incisional and ventral hernias with biological meshes in a noncontaminated field

The LAPSIS study compared open retromuscular (mesh reinforcement technique) with laparoscopic repair (mesh bridging technique) in a prospective randomized trial. Here the non-cross-linked Surgisis Gold biological mesh was compared to a classic synthetic mesh. The defect sizes were between 4 and 10 cm. The number of cases calculated for the trial was 660. The primary target criteria were recurrence rate and reoperation rate. In a letter to the editor, the study directors announced the premature termination of the trial [29]. The reasons given for premature termination were too low a recruitment rate, incomplete trial data, and a higher recurrence rate in the group with the biological meshes. Four years after starting the trial, only 265 patients, i.e., 40.2 % of the total number of cases, had been recruited. For 257 patients, a 1-year follow-up was recorded. In the laparoscopic group, a recurrence rate of 19 % was noted for the biological mesh, and a recurrence rate of 5 % was noted for the group with the classic synthetic mesh. A similar result was also observed in the group comparing open retromuscular augmentation (11 vs. 3 %). No significant differences were found for any other end points.

The conclusion drawn by the authors was that caution should be exercised when using non-cross-linked biological meshes for elective laparoscopic bridging repair of incisional and ventral hernias if the alternative use of synthetic meshes was available. Likewise, in a contaminated setting, bridging of hernia defects with this type of a biological mesh should be avoided.

In a retrospective comparative study, Cobb and Shaffer [30] compared elective laparoscopic repair of incisional and ventral hernias using a bridging technique and a composite mesh made of PP and ePTFE (Bard Composix Mesh) with the biological mesh Permacol. Permacol is a cross-linked acellular porcine dermal collagen matrix. Eighty-four procedures were carried out using Bard Composix Mesh and/or Permacol in 55 cases by a single surgeon. In the Permacol group, 15 % of procedures were conducted because of recurrences, while in the composite group 20 % of procedures were for recurrences (*p* = 0.655). Postoperative wound infections occurred in 3.3 % of cases in the Permacol group and in 2.4 % of the composite group. Mean follow-up in the Permacol group was 14 months and was 31 months in the composite group. The recurrence rate in the Permacol group was 6.6 and 1.2 % in the composite group, and as such was not statistically different (*p* = 0.17).

The authors concluded that cross-linked acellular porcine dermal collagen was a safe alternative to composite meshes made of PP and ePTFE for elective laparoscopic repair of incisional and ventral hernias using a bridging technique.

##### Laparoscopic repair of incisional and ventral hernias with biological meshes in an infected or potentially contaminated field

In a prospective trial with 116 patients, Franklin et al. [31] reported on the use of the biological mesh Surgisis in potentially or grossly contaminated fields. All procedures were performed laparoscopically with two techniques: IPOM and two-layered sandwich repair. Once the defect was totally freed of adhesions and had been closed with no. 1 Tycra sutures whenever possible, the mesh was then introduced into the abdomen and stapled securely in place with an intracorporeal stapler. Most hernia repairs were performed by the IPOM technique, except for three patients in whom the two-layered sandwich technique was performed via laparoscopic and open implantation with reinforcement with Surgisis anteriorly and posteriorly by laparoscopy. Thirty-nine procedures were carried out in an infected field and the remaining in a potentially contaminated field. Ninety-one procedures were performed concurrently with a contaminated procedure. Twenty-five presented as intestinal obstruction and 16 as strangulated hernias; 17 required small bowel resection; 29 were inguinal hernias, 57 incisional hernias, and 38 umbilical hernias. In 13 patients, more than two different hernias were repaired. The mean follow-up was 52 ± 20.9 months. Eighty-five cases were followed up for 5 years, during which 7 recurrences (6 %), 11 seromas (all resolved), and 10 cases of mild pain were identified. Six second looks were performed, and in all cases except one, the mesh was found to be totally integrated into the tissue, with strong scar tissue corroborated macro- and microscopically.

The authors concluded that the use of small intestine submucosa mesh (Surgisis) in contaminated or potentially contaminated fields is a safe and feasible alternative to hernia repair with minimal recurrence rate and satisfactory results in long-term follow-up.

### What happens to synthetic mesh after it is inserted into the body?

#### M. Fabian, B. Ramshaw MD

*Search terms:* Mesh explant (0/25), materials characterization of hernia mesh (2/6), hernia mesh explant (0/9), hernia mesh interaction (0/13), hernia mesh analysis (0/39).

The search was performed in October 2011, and a total of two unique publications were returned from this search. Both were clinical studies. A secondary search revealed an additional 10 publications pertinent to this topic. Additional information on this topic was searched for on UpToDate.

##### Statements

**Table:** 

Level 4	It appears that permanent synthetic (plastic) mesh used for hernia repair is not inert when placed in the patient’s body
Level 4	This biologic interaction is complex and the effects can be quite variable

##### Recommendations

**Table:** 

Grade D	Because there is no way to predict the biologic interaction of each patient to each available hernia mesh, the patient should be informed of potential interactions and complications. The complexity and variability of the biologic interaction would also argue against the standardization of mesh within a hospital or outpatient surgery center, allowing surgeons and patients to have options between a variety of mesh choices

##### Introduction

Hernia repair is one of the most common surgical procedures currently performed. There are over 1 million hernias repaired in the United States alone each year, and of these, over 150,000 are for incisional hernias. The vast majority of hernias are repaired with a permanent synthetic (plastic) mesh material. We are now only beginning to realize the changes that occur to the mesh and the body after placing mesh into a dynamic biologic organism [32]. The potential advantages of synthetic mesh are that mesh is accessible (easy to manufacture and maintain), consistent (materials are reproducible), durable, and cost-effective (less expensive than biological materials).

The first synthetic mesh was placed by Aquaviva in Marseille, France, in 1944, and then reported widely by Dr. Francis Usher [33, 34] in 1958. For over four decades, it was assumed that the mesh material remained inert after placement in the body. This analysis of current evidence will challenge that belief. Until recently, heavy-weight PP was by far the most commonly utilized mesh material. There are now a variety of PP-based meshes with varying densities and pore sizes as well as many meshes produced from other types of polymers. It should be noted that despite synthetic mesh reactions in the body based on current mesh explant analysis, most patients who have had mesh hernia repair have not developed mesh-related complications.

##### Research

In the late 1990s and continuing into the last decade, mesh that had been explanted for a variety of reasons was studied by a number of techniques. Histological, scanning electron microscopic, and chemical analyses, infrared spectroscopy, differential scanning calorimetry, thermogravimetric analysis, and compliance testing have all been used to test and examine synthetic mesh, mostly from prior abdominal wall hernia repair, but also after pelvic floor reinforcement [35].

The meshes have been found to undergo changes as a result of the body’s defense against foreign objects, as well as complex changes due to a chemical attack on the polymer structure [36]. There have also been many complications related to mesh hernia repair, and the result of this mesh–body interaction may be a contributing factor to these complications. Complications related to mesh interaction with the body include recurrence due to mesh contraction and/or migration, mesh erosion into viscera and/or through skin, chronic pain, functional issues resulting from lack of mesh compliance, acute and delayed mesh infection, acute and chronic inflammatory reactions including chronic active seroma, and rare systemic symptoms, such as flulike symptoms, potentially related to synthetic mesh. The variety of methods used to study mesh after explantation from the body are now presented.

##### Histology

At the cellular level, the body will attempt to wall off, digest, or expel the foreign material. Cellular immunity is critical for survival, yet it creates problems in some (but not all) hernia patients. PP seems to have the greatest inflammatory reaction of the synthetic meshes, but this appears to decrease over time [37].

Neutrophils, lymphocytes, macrophages, and foreign-body giant cells are stimulated upon injury (surgery) and implantation of mesh material. These cells release enzymes and oxidants to degrade the foreign body—in this case, the mesh [38]. Study of mesh has shown oxidative breakdown in addition to encasement with inflammatory cells. Lymphocytes and foreign-body giant cells are present, and these can bathe the mesh in a continuous environment of oxidants while progressively encasing the mesh in a fibrous scar that can become increasingly rigid. This may be a contributing factor to chronic, and in some cases debilitating, pain [39].

The foreign-body response has been classified as having four distinct phases: acute inflammation, chronic inflammation, foreign-body reaction with development of granulation tissue, and fibrosis [38]. Heavy-weight PP meshes exhibit more collagen deposition and fibrosis, while lightweight meshes exhibit minimal fibrotic tissue with better neovascularization around the mesh [40].

The oxidants released by lysosomes can create superoxide anions as well as hydrogen peroxide and hypochlorous acid [41]. PP has been shown to undergo chain scission, and overall degradation with fissures, micro cracks, build-up of hydroxyl and carbonyl groups on the surface of the material, changes in thermal properties, and changes in mechanical properties such as embrittlement and reduced compliance.

There has also been discussion that the meshes generally shrink as a result of the above-listed changes. However, this contraction or shrinkage appears to be a complex and irregular process. Coda et al. [42] studied multiple types of mesh and discovered that the explanted mesh pore sizes could have expanded up to 58 % as well as shrunk by 40 %.

##### Scanning electron microscopy

Most micrographs have demonstrated changes to the PP mesh that include micro cracks in the transverse direction, as well as peeling of the top layer of fibers [40]. Other changes included superficial or deep flaking and fractures in the threads of varying lengths and depths [35]. Interestingly, polyethylene terephthalate did not appear degraded in two separate studies [35, 43]. These findings are contrary to other reports on degradation of vascular grafts, and much more study of this complex biologic interaction is needed.

##### Fourier transform infrared spectroscopy

Fourier transform infrared spectroscopy is a spectroscopic technique widely used to facilitate determination of chemical functional groups by their absorption frequency. In 2010, two studies examined multiple types of mesh [35, 44]. These studies found that in virtually all types of synthetic mesh, peaks representing hydroxyl and carbonyl groups were present. This has even been noted in ePTFE, one of the meshes thought to be the least affected by alterations.

This indicated a chemical breakdown of the “inert” mesh that has potential implications for the strength of the polymer. Many of the hydrocarbon propylenes depend on van der Waals forces, and the alteration of the chemical groups can weaken these bonds. The overall effect may explain the changes in mesh seen in the tests mentioned below.

##### Differential scanning calorimetry

This test measures melting temperature and heat of fusion in materials, and was tested in a variety of explanted meshes. This showed a shift toward lower melting temperature and broader melting peak. The clinical implication is not clear but demonstrates a change in the physical properties of the mesh.

##### Thermogravimetric analysis

This measures weight loss of the material versus a pristine piece of mesh. This was lower for all mesh tested. This is now intuitive, as the material has been assaulted by the body, exposed to oxidative forces, and broken down chemically. This would also explain the mechanical failure of some lightweight meshes, which have been designed to lessen the host response with fibrosis and scarring, but sacrifice strength to achieve this.

##### Compliance testing

This measures the mean value of work to bend the mesh in half using a constant force. Nearly all materials tested, even after removing all organic material, required more work and were less compliant than the pristine control mesh. However, this compliance testing revealed tremendous variability between explant samples [39, 40].

##### Summary

Since the early 1990, a diverse group of individuals, including materials engineers, chemical engineers, pathologists, device company representatives, and surgeons have made early attempts to begin to understand the changes that occur after mesh implantation in human beings. Animal experiments have not been able to show the long-term consequences of foreign-body implantation into biologic organisms. The host response is variable, and we have only begun to realize the individualization that will be needed to find the best mesh for a particular cluster of individuals. There will likely be groups of patients who will have a better outcome with certain types of mesh as well as certain groups of patients who will be at risk for increased mesh-related complications with certain types of mesh. To attempt to define these groups, an evolved understanding of clinical research based on principles of complex systems science will likely be needed.

## Section 8: Hernia prophylaxis

### Open abdominal surgery and stoma surgery. Indications for prophylactic mesh implantation and risk-reduction strategies

#### T. Simon, D. Berger

*Search terms:* (indic* AND prophyl* AND mesh)) OR (“Hernia, Ventral/prevention and control” [Mesh] OR “Hernia/prevention and control”[Mesh] OR “incisional hernia” AND (prevention OR prophyl*) OR “abdominal wall hernia” AND (prevention OR prophyl*) OR “Hernia, Abdominal/prevention and control”[mesh]) OR “hernia prevention” OR “hernia prophylaxis” OR “prophylactic mesh” OR “mesh implantation” OR (mesh AND “risk reduction” [tiab]) AND (randomized controlled trial [pt] OR controlled clinical trial [pt] OR randomized [tiab] OR placebo [tiab] OR clinical trials astopic [mesh: no exp] OR randomly [tiab] OR trial [ti] NOT (animals [mh] NOT humans [mh])).

A systematic search was performed of PubMed, Medline, Cochrane, Study register, and relevant journals and reference lists including publications until June 6, 2012.

The search produced 895 articles; with RCT (randomized controlled trial) filter 128 and Systematic Review filter, 39 papers resulted. Regarding open abdominal surgery and the indication for prophylactic mesh, six relevant publications were identified, whereas two level 2a, one level 2b, one level 3, three level 4, and one experimental study were stratified. For stoma surgery and indications for prophylactic mesh, four systematic reviews and one protocol for a Cochrane review were identified. There were 21 publications dealing with risk-reduction strategies to prevent incisional hernias.

##### Statements

**Table:** 

Level 2	Prophylactic mesh placement reduces the rate of incisional hernia in risk groups with morbid obesity or aortic aneurysm
Level 1	Prophylactic mesh placement in primary stoma formation reduces the rate of parastomal hernia without increasing morbidity, although this is based on small patient populations
Level 2	There is no relevant difference between midline and transverse incisions regarding the incidence for incisional hernia formation
Level 1	Fascia closure with a continuous suture technique using slowly resorbable suture material reduces the incidence for incisional hernia after elective median laparotomy significantly
Level 4	Achieving a suture length to wound length ratio of 4 or more significantly reduces the incidence of incisional hernia after midline incision

##### Recommendations

**Table:** 

Grade B	A prophylactic mesh should be placed after open abdominal surgery in risk groups with morbid obesity or aortic aneurysm
Grade A	A prophylactic mesh should be placed at the primary stoma operation, although this is based on small patient populations
Grade B	The access to the abdominal cavity can be reached by either by a transverse or a midline incision, based on the surgeon’s preference with respect to the patient’s disease and anatomy
Grade A	After elective median laparotomy, the fascia should be closed with a continuous suture technique using slowly resorbable suture material
Grade D	A suture length to wound length ratio of 4 or more should be accomplished when closing the abdomen

##### Introduction

The incidence of incisional hernias has been reported between 5 and 20 %, causing it to be the most common surgical complication after laparotomies [45–49]. With this burden of disease for patients with complications after surgical repair, the increasing risk for recurrence [50], and the economic consequences, the need for studies dealing with risk-reduction strategies is obvious. For parastomal hernias, defined as an “incisional hernia related to an abdominal wall stoma” [51], the incidence is up to 48 % or greater. Besides the technical aspects of wound closure and stoma formation, the use of biological and synthetic meshes for prophylaxis of incisional and parastomal hernia has been the subject of several studies.

##### Indication for prophylactic mesh implantation for open abdominal surgery

In addition to the high occurrence rates of hernia after laparotomy, several studies have identified two major risk groups with even higher rates. For patients with an abdominal aortic aneurysm (AAA), a meta-analysis has shown a 5-fold increased risk of incisional hernia development [52]. It is presumed that a systemic connective tissue disorder may be responsible for the high rates of incisional hernias in these patients [53, 54].

The second risk group for postoperative wound complications, especially the development of incisional hernia after laparotomy, are morbidly obese patients [55]. Other studies reported rates of postoperative hernias for obese patients of up to 50 % [56, 57].

The objective of this study was to find evidence for the use of prophylactic mesh to minimize the risk for incisional hernia. A small case series with a prophylactic mesh placed in the preperitoneal space after open AAA repair resulted in a low rate of incisional hernia after a median follow-up time of 47 months [58]. A well-conducted RCT with a 3-year follow-up showed a significant reduction of postoperative incisional hernia after AAA repair without increasing the rate of complications, although patients with previous abdominal surgery were not excluded [59].

The first RCT with long-term results after prophylactic mesh to prevent incisional hernia in obese patients did not reveal an advantage for the mesh group [60]. However, it must be noted that the study group used a resorbable polyglactin mesh.

A case series with 60 patients undergoing gastric bypass surgery and a midline incision closure with a nonresorbable PP mesh demonstrated an effective prevention of incisional hernia [61]. The same group conducted a RCT for the prophylactic use of a mesh with a mean follow-up of 28 months and found an incidence of over 20 % incisional hernia in the nonmesh group and none in the mesh group [62]. The strength of this research was weakened as a result of the lack of a blinded arm and the small number of patients. A prospective study without randomization of 100 high-risk patients (including neoplastic pathology, age over 70 years, respiratory failure, malnutrition, obesity, and smokers) also showed a significant reduction of the development of incisional hernia with the use of a prophylactic PP mesh [63] (Table 2). In a two-institution nonrandomized prospective trial in which a biologic mesh was applied to one patient group compared to the nonmesh group at the other institution after gastric bypass, a reduction of the incidence of incisional hernia in the mesh group was revealed [64]. All these RCT studies show substantial weaknesses regarding the study design and methods, resulting in downgrading of their evidence level. An ongoing double-blind randomized controlled multicenter trial, PRIMA, includes both high-risk groups with patients being operated for AAA or other median laparotomies with a body mass index (BMI) of over 27 kg/m^2^ [65]. The recruitment process is accomplished, and the publication of the trial is awaited.

**Table 2:** RCT studies for open abdominal surgery

Study	No. of patients	Population	Comments
Pans [60]	288	Obese	Resorbable mesh
Gutierrez de la Pena [63]	100	Mixed	Unclear randomization
Strzelczyk [62]	74	Obese	Unblinded
Bevis [59]	80	AAA	Includes patients with previous laparotomies

*RCT* randomized controlled trial, *AAA* abdominal aortic aneurysm

##### Indication for prophylactic mesh implantation for stoma formation

The repair of parastomal hernias results in high complication and recurrence rates [51, 66, 67]. Although the approach of laparoscopic repair of parastomal hernias with intraperitoneal meshes has shown better results, with recurrence rates under 12 %, the complication rates are still high [68, 69]. Hence, the prevention of the parastomal herniation by placing a prophylactic mesh in the abdominal wall at the primary operation has been subject of several studies.

Bayer et al. [70] published the first study of prophylactic mesh implantation to prevent paracolostomy hernia formation in 1986. The first RCT was stopped by the authors after the inclusion of 21 patients because of a significant difference between the groups [71]. After a mean follow-up of 24 months, 13 of 27 patients in the nonmesh group showed a parastomal hernia, whereas in the mesh group only one patient had a hernia. A systematic review including the above study and some case reports concluded that the preliminary results of a prophylactic mesh in stoma formation were promising [72]. In the randomized study of Hammond et al. [73], only ileal loop stomas were included and reinforced with a biologic mesh in the treatment group; only 10 patients were in each group. The results of the 5-year follow-up published by Janes et al. [74] in 2009 confirmed the initial results of the above-mentioned trial with a significant reduction of hernia rates. A third RCT, conducted by Serra-Aracil et al. [75], included 27 patients scheduled for permanent end colostomy surgery in each group. This study had a median follow-up of 29 months. A hernia rate of 40.7 % was observed in the nonmesh group versus 14.8 % in the mesh group. This was significantly lower (*p* < 0.05) and was not associated with any mesh-related complication.

Three other systematic reviews and one Cochrane review have been published; the latter includes the same three RCTs [76–79]. A total of 128 patients (mesh 64, nonmesh 64) were eligible and analyzed in the latest review by Shabbir et al. They concluded that despite a small patient population, it could be demonstrated that the use of a prophylactic mesh at the primary stoma operation reduced the incidence of parastomal herniation with a very low morbidity. A large RCT to focus on mesh material and anatomic location is needed to confirm these findings.

##### Risk-reduction strategies

Against the background of high incidences of incisional hernia after laparotomies, efforts to reduce the risk should be taken [80]. Besides patient-related risk factors, technical aspects such as suture material, suture length, suture technique, and access to the abdominal cavity are subjects of several studies.

##### Access to the abdominal cavity: midline versus transverse incision

The most commonly used incisions to gain access into the abdominal cavity are midline or transverse incisions. Related complications and relevant outcomes are incisional hernia, wound infection, and pulmonary complications. In 2005, a systematic review of the Cochrane Collaboration showed a slightly advantage for the transverse incision with respect to postoperative pain and a negative influence on pulmonary function [81]. Because of inadequate blinding, unclear randomization procedures, and small sample sizes of the underlying study populations, these results are not conclusive. Comparing the one-sided transverse incision with midline incision for open cholecystectomies, the RCT conducted by Halm et al. showed significantly fewer incisional hernias for transverse incisions for this specific indication [82]. Another prospective randomized trial revealed an incidence of incisional hernia of over 90 % for the midline incision compared to 40 % for a transverse incision for aortic aneurysm repair [83]. However, the results must be viewed critically because there were only 22 patients in the midline incision group and 15 patients in the transverse incision group.

A randomized controlled double-blind equivalence trial, POVATI, comparing both incisions found no significant difference regarding pain, pulmonary complications, and incisional hernia development after 1 year [84]. However, significantly more wound infections occurred in the transverse incision group.

##### Closure technique

There is no consensus in the surgical community regarding wound closure techniques after laparotomies as shown in a cross-sectional cohort study [85]. Several RCTs are available that focus on this issue, and five systematic reviews pooled the available data without defining homogenous study populations and follow-up [47, 86–89].

With precisely defined study populations and follow-up periods, the INLINE systematic review and meta-analysis revealed the highest available evidence [90]. The risk for incisional hernia after elective median laparotomy is significantly lower if the fascia is approximated with a continuous suture technique using slowly absorbable suture material. For emergency settings, the results of the randomized controlled multicenter trial, CONTINT, must be awaited [91].

Technical aspects of suture techniques are suture length and stitch width. In a prospective trial with 363 patients after midline laparotomy in elective and emergency settings, Israelsson and Jonsson [92] found an overall incidence of incisional hernia of 18.7 % after 12 months. When stratified for a suture length to wound length ratio, the group with a ratio below 4 had an incisional hernia rate of 23.7 %, whereas in the group with a ratio of 4 or more, the incidence was 9.0 % (*p* > 0.001). These results could be confirmed in several cohort studies [93–95]. The subject of several studies and ongoing trials is the question of stitch technique [96, 97]. In an experimental study, wound closure with stitches placed 3 mm from the wound edge was stronger compared to those placed at least 10 mm [98]. Another experimental study found similar results [99]. In an RCT with 381 patients in the long-stitch group and 356 patients in the short-stitch group, the latter showed an incidence of incisional hernias 12 months after operation of 5.6 % compared to 18 % in the long-stitch group (*p* > 0.001) [100]. Additionally, long stitch length was identified as an independent risk factor for surgical site infection The authors recommended the use of a 150 cm long 2-0 (USP) suture with a small needle to accomplish a suture length to wound length ratio of at least or more than 4. The small needle is suggested to prohibit be ability to achieve large bits of tissue.

Gaining 1b evidence is the question of whether the “small bite” stitch technique is superior to the commonly used “big bite” technique in terms of costs and effectiveness. To further investigate this claim, a randomized controlled multicenter trial, STICH, was initiated and is currently active [101].

## Section 9: Technique—special issues

### Is laparoscopic preperitoneal ventral and incisional hernia repair possible?

#### W. Reinpold

*Search terms:* “endoscopic preperitoneal repair” or “laparoscopic preperitoneal repair” or “endoscopic sublay repair” or “laparoscopic sublay repair” and “ventral hernia” or “incisional hernia” or “abdominal wall hernia” or “umbilical hernia.”

Searches were performed in PubMed, Medline, Embase, Br J Surg Database, Science Citation Index, and the Cochrane database. Twelve publications were included in this review.

##### Introduction

Currently, laparoscopic IPOM [102] and open sublay repair, first described by Rives et al. [103], are the most frequently used techniques for the treatment of primary and incisional abdominal wall hernias. In the literature, laparoscopic IPOM repair is associated with fewer infections and wound-healing complications compared to open mesh repairs [102]. In contrast to all other laparoscopic procedures, acute and chronic pain does not seem to be reduced after laparoscopic IPOM operations. The IPOM technique is performed with expensive composite meshes, the bowel-facing surface of which is covered with an adhesion preventing material or pure ePTFE. IPOM meshes have to be fixed securely with transfascial sutures, staples, or clips, which carry the risk of adhesion and/or acute and chronic postoperative pain. The long-term safety of IPOM meshes has not been proven in clinical studies.

Other potential disadvantages of the laparoscopic IPOM repair are as follows: in most cases, the hernia sac stays in situ, the defect is bridged, and the abdominal wall is not reconstructed; adhesions between the viscera and abdominal wall have to be taken down; and severe complications such as bowel injury appear to be more common.

For a further improvement of abdominal wall hernia repair, the advantages of the sublay repair and laparoscopic IPOM repair should be combined. The question is, can a preperitoneal ventral and incisional hernia repair be achieved with fewer complications and better long-term results?

The conclusions and recommendations on laparoscopic preperitoneal ventral and incisional hernia repair are based on a systematic review of the literature and a consensus conference on guidelines for the laparoscopic treatment of ventral and incisional hernias held in October 2011 Suzhou, China, during the fifth meeting of the International Endohernia Society (IEHS).

##### Statements

**Table:** 

Level 4/5	Laparoscopic transperitoneal and total extraperitoneal preperitoneal/sublay repair are surgical options for the treatment of small- and medium-sized ventral and incisional hernias (EHS classification W1 and W2)
	Both techniques allow the implantation of large standard synthetic prostheses
	These procedures are technically demanding and have longer operating times than open preperitoneal/sublay repair and laparoscopic IPOM repair but do not require barrier meshes
	Laparoscopic preperitoneal repair combines the advantages of open preperitoneal repair and laparoscopic IPOM technique: small incisions and extraperitoneal mesh position
	Complication rates are low

##### Recommendations

**Table:** 

Grade C	Laparoscopic transperitoneal and total extraperitoneal preperitoneal/sublay repair are surgical options for the cure of small- and medium-sized ventral and incisional hernias (EHS classification W1 and W2) if expertise is present
Grade D	Especially in the lower abdomen, laparoscopic transperitoneal or extraperitoneal preperitoneal abdominal wall hernia repair can be considered if the required expertise is available

##### Laparoscopic preperitoneal abdominal wall hernia repair

There are only few literature reports on laparoscopic preperitoneal abdominal wall hernia repair [104–113]. As in inguinal hernia repair (transabdominal preperitoneal [TAPP] and totally extraperitoneal [TEP] repair), the laparoscopic preperitoneal mesh repair of ventral and incisional hernias can be performed via a transperitoneal or totally extraperitoneal approach. The mesh may be separated from the abdominal cavity by the peritoneum only, the posterior rectus sheath, and peritoneum or the urinary bladder.

##### Laparoscopic transperitoneal preperitoneal mesh repair of ventral and incisional hernias (TAPP)

In the literature there are 47 cases of laparoscopic transperitoneal preperitoneal abdominal wall hernia repair reported, mainly in the lower abdomen [104–110]. Small- and medium-sized suprapubic, umbilical, lumbar, epigastric, and port-site hernias have been operated on via laparoscopic transperitoneal preperitoneal mesh repair. In the lower abdomen, a modified TAPP technique can be used, especially for the treatment of spigelian hernias [105, 107]. Since 2003, the author’s working group has performed 142 TAPP operations of primary and incisional epigastric, umbilical, combined umbilical and epigastric, lateral abdominal wall, spigelian, and port-site hernias with the implantation of standard PP meshes.

In a prospective cohort trial with a control group, Schröder et al. (under review) report on a three-port laparoscopic transperitoneal sublay repair technique via the left flank. In 43 small- and medium-sized ventral and incisional hernias, medium- and large-sized pieces of standard PP mesh (15 × 15 cm up to 30 × 20 cm) were implanted. The follow-up rate was 92 % with a median of 16 months. Compared to the open sublay repair group, there was less acute pain and the hospital stay was shorter. However, operating time was longer in the laparoscopic group. There were no differences in chronic pain and discomfort. In both groups, no recurrences or wound infections were noted. The authors concluded that laparoscopic transperitoneal sublay repair is a safe and effective method for the treatment of small- and medium-sized primary and incisional abdominal wall hernias combining the advantages of open sublay and laparoscopic IPOM repair.

##### Endoscopic total extraperitoneal preperitoneal abdominal wall hernia repair

Three publications describing 17 cases of endoscopic total extraperitoneal mesh repair of abdominal wall hernias (abdominal wall TEP) were found [111–113]. Miserez and Penninckx [111] published 15 cases of abdominal wall TEP of the rectus compartment in 2002. There are two case reports of spigelian hernia TEP repair.

Reinpold et al. (oral presentation, EHS Congress, Istanbul, 2010) developed a transhernial single-port TEP technique for the treatment of primary and incisional abdominal wall hernias. The hernia sac and midline defect are dissected through a 3- to 4-cm incision. The extraperitoneal space around the defect is enlarged by separation of the peritoneum from the fascia. Large hernia sacs are removed and defects of the peritoneum are closed. A single port with three 5-mm trocars is inserted into the defect. Using a pneumoperitoneal pressure of 10 mmHg, the circumference of the defect is dissected endoscopically. A standard PP mesh is inserted in the sublay position and fixed with sutures or tacks at the lateral border. Alternatively, a self-fixating mesh can be used. The midline defect is closed via the port incision. Twenty-four patients with an average defect size of 17 cm² (range, 9–61 cm^2^) were operated on. The average mesh size was 232 cm^2^ (range, 96–600 cm^2^). Pain medication was stopped in all patients after a maximum of 4 days. Two small retromuscular hematomas were treated conservatively. After an average follow-up of 8 months (range, 2–15 months), there was no chronic pain, recurrence, or infection.

##### Conclusion

Laparoscopic preperitoneal abdominal wall hernia repair via the TAPP and TEP techniques in small- and medium-sized primary and incisional abdominal wall hernias is feasible and has minimal morbidity. The advantages are: (1) access causes minimal trauma; (2) standard mesh with minimal fixation can be used; (3) the abdominal cavity is only minimally entered; (4) the hernia sac is removed from the abdominal wall; and (5) the hernia defect is closed and the abdominal wall is reconstructed anatomically. However, the technique is more demanding and takes longer to perform than standard procedures.

### The role of endoscopic component separation in the treatment of large abdominal wall hernias

#### W. Reinpold

*Search terms:* “endoscopic component separation” or “laparoscopic component separation” and “ventral hernia” or “incisional hernia” or “abdominal wall hernia.”

Search databases used were PubMed, Medline, Embase, Br J Surg Database, Science Citation Index, and the Cochrane database. Seventeen publications describing 128 cases of endoscopic component separation (ECS) were identified. The conclusions and recommendations on ECS are based on this systematic review as well as on a consensus conference on guidelines for the laparoscopic treatment of ventral and incisional hernias held in October in Suzhou, China, during the fifth meeting of the IEHS.

##### Introduction

Very large incisional hernias with a horizontal defect of more than 10 cm are a challenge in abdominal wall hernia surgery. In many of these giant incisional hernias, standard open techniques and the laparoscopic IPOM repair are insufficient. The defect closure with reconstruction of the linea alba can often only be achieved with the open component separation (OCS), as described by Ramirez et al. [114] in 1990. The OCS gives an abdominal wall release of 10–15 cm on every side but requires an extensive dissection of subcutaneous tissue of the abdominal wall with division of the deep perforating vessels. This leads to a high rate of wound infections and wound-healing problems [115–119].

##### Statements

**Table:** 

Level 3	The ECS is feasible with low morbidity
	The ECS can be combined with lap IPOM, open IPOM, open sublay, and open onlay technique in complex hernias
	Abdominal wall release after ECS is less extensive than after OCS
	There are fewer wound infections and wound healing problems after ECS compared to OCS
Level 4	The question whether the lateral compartment should be augmented with mesh remains unresolved

##### Recommendations

**Table:** 

Grade C	In large and very large ventral and incisional hernias, the ECS can be considered in combination with open or laparoscopic mesh techniques if the surgeon is able

The ECS can be combined with other open or laparoscopic procedures [116–121]. Losanoff et al. [122] were the first to report on endoscopic-assisted component separation in 2002. In 2007, Rosen et al. [115] published a retrospective study of seven patients who underwent an ECS for abdominal wall reconstruction during the resection of an infected prosthetic material in complex abdominal wall hernias. The technique of ECS as described by Rosen et al. [115] is as follows: below the costal margin and lateral of the rectus compartment, a bilateral 15-mm skin incision is created and a 10-mm balloon dilator is inserted. Blunt dissection is performed of the avascular space between the external and internal oblique muscle. Two trocars are inserted, CO_2_ is insufflated, and further dissection is done of the space under camera vision. The fascia of the external oblique muscle is vertically incised lateral to the rectus compartment from the costal margin to the inguinal area.

The residual defect size after the removal of all prosthetics was 338 cm^2^ (range, 187–450 cm^2^). ECS enabled tension-free primary fascial reapproximation in all patients. There was one superficial surgical site infection. After an average follow-up period of 4.5 months, no recurrences were identified.

Harth and colleagues [116, 117] reported a retrospective study on 32 ECS compared to 22 OCS. Open component separation had a 41 % major wound morbidity rate compared to 19 % in the endoscopic group (*p* = 0.07). Hernia recurrences rates were similar (open, 32 %; endoscopic, 27 %; *p* = 0.99). Hospital length of stay was 11 days after OCS versus 8 days after ECS (*p* = 0.09). The median mesh costs differed significantly between ECS and OCS ($733 vs. $8415; *p* = 0.05). The authors concluded that there were significantly fewer wound complications after ECS and similarly high rates of recurrence.

These findings were confirmed by others [118–121] and by our working group (in preparation). We performed a bilateral ECS combined with an open sublay repair in 23 patients with large ventral incisional hernias with an average defect size of 210 cm² (range, 72–454 cm²). Complete reconstruction of the linea alba was achieved in 18 patients. The abdominal wall release on each side was 2–6 cm. All patients received a total rectus compartment sublay repair with large-pore PP mesh. The average follow-up was 21 months (range, 4–37 months) in 19 patients. Complications that were noted included three hematomas that resolved spontaneously, three cases of lateral abdominal wall bulging, and one superficial wound infection that did not require a reoperation.

The ECS can be combined with lap IPOM, open IPOM, open sublay, and open onlay technique in complex hernias. The abdominal wall release after ECS is less extensive than after open OCS [117–120]. There are fewer wound infections and wound-healing problems after ECS compared to OCS [116–121]. The question whether the lateral compartment should be augmented with mesh is unresolved. No long-term data are available. Further studies for the assessment of the ECS are necessary.

### Laparoscopic parastomal hernia repair

#### S. Morales-Conde

##### Introduction

Parastomal hernias are the most frequent complication that occurs after a stoma is created. The exact incidence is not easy to establish because this problem is underestimated by both patients and physicians. The reported incidence rate ranges from 2.8 to 50 % [123] and appears to be directly related to the length of follow-up. Loop ileostomy has the lowest risk (0–6.2 %), followed by end ileostomy and loop colostomy, which has a similar risk of 28–30 %. End colostomy carries the highest risk for parastomal hernia of more than 50 %. Even though most hernias occur within the first 2 years after stoma construction, the risk of herniation extends up to 20 years [124].

Although many risk factors have been related to the development of a parastomal hernia, waist circumference, patient age, and stoma size are independent risk factors for the development of a parastomal hernia after a permanent colostomy [124, 125]. Parastomal hernia is asymptomatic most of the time, but it may be associated with serious complications such as strangulation and perforation; elective repair is thus mandatory for many selected cases. The diagnosis is generally made by clinical examination. The computed tomographic scan is very useful to determine the content of the hernia, the size of the defect, and the existence of a concomitant hernia at the midline or other incisions.

Many different surgical techniques have been described for the treatment of parastomal hernias. Nonmesh techniques are known to have a high rate of recurrence (46–100 %) and should generally not be performed [126, 127] because the mesh techniques offer significantly better results. Meshes could be placed as an onlay or sublay through a local incision close to the stoma. However, these techniques have an incidence of wound infection of up to 30 % [128, 129]. The underlay or IPOM position not only offers a decreased rate of wound infections but also affords the opportunity to repair a concomitant incisional hernia, if present. The laparoscopic approach achieves the advantages of a minimally invasive approach with the low incidence of infection and recurrence rate that the intra-abdominal placement of a mesh offers.

*Search terms:* Laparoscopic, laparoscopy, paracolostomy, colostomy, para-colostomal, colostomal, para-ileostomy, ileostomy, ileal conduit, urostomy, hernia, defect, repair, closure, reconstruction.

A Medline search was performed until November 2011. A total of 73 papers were identified, but only 27 were relevant to the review. In the final analysis, there were no articles with level of evidence 1, 2, or 3a, and only three papers with level of evidence 3b [131–133], 16 with level of evidence 4 [134–149], and 8 with level of evidence 5 [150–155]. One of the level 3b articles compared two of the different techniques used to perform the repair of parastomal hernias by laparoscopy [133], while the other two compared the open approach versus laparoscopic techniques [131, 132]. One of these two studies was of very poor quality because the authors compared the laparoscopic approach with a wide variety of open techniques, including no-mesh and mesh techniques [132]. Eight of the studies with level 4 evidence were cases series, each with fewer than 10 cases.

#### Is the laparoscopic approach to parastomal hernia repair superior to the open approach?

##### Statements

**Table:** 

Level 3	Laparoscopic repair of parastomal hernias can be performed safely
Level 4	The rate of recurrences after laparoscopic repair of parastomal hernias are lower than the open approach

##### Recommendations

**Table:** 

Grade B	Laparoscopic repair of parastomal hernia should be considered a safe alternative to the open approach
Grade C	Laparoscopic parastomal hernia repair is a valid alternative option to open repair because its rate of recurrence appears to be lower than the open approach

##### Discussion

Open suture repair of the fascial defect of a hernia or a stomal hernia are both associated with high morbidity and an unacceptably high recurrence rate. Consequently, this repair is no longer recommended for routine use for either hernia. Primary closure of the aponeurosis at the hernia site, either via a peristomal approach or through a midline incision, is a simple procedure, but it carries a recurrence rate of 38–100 %. Stomal relocation may result in a zero recurrence rate at the same hernia site, but the risk of a parastomal hernia after the new stoma formation is as high as 46 %. In addition, an incisional hernia at the previous colostomy closure site may also occur. For this reason, the use of PP meshes has been applied to this repair, either to reinforce a suture repair or to bridge the fascial gap. The recurrence rate with this open technique is still on the order of 20–33 % [124]. Additionally, complications related to PP meshes have been described, such as obstruction, fistulization, or mesh erosion [145]. Meshes can be placed in different anatomic positions: during the onlay repair, the mesh is subcutaneously placed and fixed to the fascia of anterior rectus muscles and to the aponeurosis of the external oblique abdominal muscle; a retromuscular technique indicates that the prosthesis is placed dorsally to the rectus muscle and anteriorly to the posterior rectus sheath; with an intraperitoneal position, the mesh is placed intra-abdominally after being fixed to the peritoneum. Two techniques are used to repair parastomal hernias with an intraperitoneal placement of a prosthesis: the Sugarbaker technique and the keyhole technique. In 1985, Sugarbaker [156] described a new technique for parastomal hernia repair through a midline laparotomy; the bowel was lateralized passing from the hernia sac between the abdominal wall and the prosthesis, which was sutured to the fascial edge covering the opening.

The laparoscopic approach involves minimally invasive access to the abdominal cavity and intraperitoneal placement of prosthetic material with or without narrowing the defect. Similar to the open intraperitoneal mesh repair, the Sugarbaker technique, the keyhole technique, and a combination of the two described by Berger and Bientzle [142] and known as the sandwich technique are used. The laparoscopic approach makes a peristomal incision unnecessary and also decreases the potential risk of mesh infection. Published series on laparoscopic mesh repair of parastomal hernia, however, are few, with relatively short follow-up.

There are two studies with level 3b evidence that compare the open approach with the laparoscopic techniques to repair parastomal hernias. Both papers are retrospective studies, but the one conducted by McLemore et al. [132] includes in the open group cases in which a suture repair was performed together with mesh techniques and relocation of the stoma (associated with differing rates of recurrence). On the other hand, this author also included a laparoscopic group of cases performed after the keyhole and the modified Sugarbaker technique. Both techniques are associated with a different rate of recurrence.

The most important message coming from the other study with level 3b evidence, conducted by Pastor et al. [131], is that the morbidity rate of the laparoscopic approach was 15 %, while the complications after the open approach were up to 33 %. This same study showed a lower recurrence rate after the laparoscopic approach than after the open technique (33 vs. 53.8 %). It was noted that the time of the follow-up was different (13.9 vs. 21.4 months) in the two groups. Therefore, it was noted that the rate could increase with time.

In order to draw conclusions regarding recurrence, it is best to analyze the studies with level 4 evidence. Even though there are some cases series with high recurrence rates of up to 56 % [146], most of the studies report a recurrence rate below 10 % with the laparoscopic approach. This represents a much better recurrence rate than the results of the open approach. At present, none of the methods of open or laparoscopic mesh repair has proved superior. In spite of this, laparoscopic repair has gained increasing acceptance.

#### Does laparoscopic parastomal hernia repair have similar results when compared to laparoscopic ventral hernia repair?

##### Statements

**Table:** 

Level 4	Operative times for parastomal hernia repair are longer than a LVHR because the technique is more difficult, especially because of a more difficult process of adhesiolysis
	Intraoperative complications during laparoscopic repair of parastomal hernias are more frequent than during standard LVHR
	A high percentage of parastomal hernias are associated with an additional midline incisional hernia, which makes the surgical procedure more complex
	The rates of both recurrence and morbidity are higher after laparoscopic parastomal hernia repair than after LVHR

##### Recommendations

**Table:** 

Grade C	A laparoscopic approach of parastomal hernias should be considered a difficult technique with longer operating time, more intraoperative complications, and more difficult adhesiolysis than standard LVHR
	Results of laparoscopic repair of parastomal hernias could not be compared to the general results of LVHR because the rates of recurrence and morbidity are higher
	Laparoscopic repair of parastomal hernias is a more complex technique because a concomitant midline hernia present in a high percentage of patients must also be repaired

##### Discussion

If we compare the results of laparoscopic repair of parastomal hernias with the data published with level 1a evidence on LVHR, we observe that the surgical time associated with the laparoscopic repair of parastomal hernia is longer and the morbidity higher [157, 158]. These data show that this technique seems to be more difficult than standard LVHR because of the presence of more dense adhesions and the frequent concomitant midline incisional hernias. It is also more challenging to separate the adhesions of the ostomy itself from the omentum and other intestines. Additionally, the rate of infection of a LVHR is close to zero, but the rate of postoperative infection or other late mesh-related complications is higher after laparoscopic parastomal hernia repair. It has been reported to be as high as 7–9.5 % [137, 141]. The conclusion of this comparison is that laparoscopic parastomal hernia repair is a more complex procedure than standard LVHR and should be performed by an expert surgeon.

A comparison between the data observed in different studies with different levels of evidence for the LVHR reveals that the overall recurrence rate of this technique is lower than the recurrence rate observed after laparoscopic repair of parastomal hernias. This can be explained by the complexity of the latter technique and by the relative early stage of its development; the best technique—keyhole, Sugarbaker, or sandwich—remains to be defined. There is an increasing amount of evidence that laparoscopic mesh repair is feasible and has a promising potential in the management of parastomal hernia.

#### Which is the best laparoscopic technique for repair of parastomal hernias?

##### Statements

**Table:** 

Level 3b	Laparoscopic repair of parastomal hernias using a pure ePTFE mesh is associated with better results than the keyhole technique
Level 3b	The laparoscopic modified Sugarbaker technique or the sandwich technique results in fewer recurrences than the keyhole technique
Level 4	The results of the three main laparoscopic technique used to repair parastomal hernias (Sugarbaker, keyhole, and sandwich) are similar

##### Recommendations

**Table:** 

Grade B	Laparoscopic repair of parastomal hernia using the modified Sugarbaker technique should be recommended when a pure ePTFE mesh is used
	Although the keyhole technique has a lower recurrence rate compared to the Sugarbaker technique, this could be related to the type of mesh because series not using a pure ePTFE mesh show similar recurrence rates as the Sugarbaker technique with this type of mesh
Grade C	None of the technique described in the literature—Sugarbaker, keyhole, or sandwich—is superior
	Although there is only one series with the sandwich technique (using two meshes), this technique can be considered a safe alternative to the keyhole or Sugarbaker techniques
	The same laparoscopic technique can be performed for a hernia occurring with a colostomy, ileostomy, or urostomy, or due to an ileal conduit

##### Discussion

Laparoscopic parastomal hernia repair has become a viable option to overcome the challenges that face the hernia surgeon. Most series suffer from a small sample size, and controlled trials are lacking. These limited data make it difficult to draw firm conclusions. Two laparoscopic techniques have emerged: the use of a mesh with a slit and a central keyhole and a mesh without a slit. The latter is often termed the modified Sugarbaker. A third option, the sandwich technique, has been also been described and consists of a combination of both techniques.

Published series, however, are observational and often have a short follow-up. There is only one comparative study with level 3b of evidence: that of Muysoms et al. [133]. In this study, the authors show that the modified Sugarbaker technique offers significantly lower recurrence rates than the keyhole technique (72.7 vs. 15.4 %), although the follow-up of those cases performed following the Sugarbaker technique is shorter than the rest of the cases (30.7 vs. 14 months). Together with this study, Hansson et al. [68] showed a very low recurrence rate (18 %) when the keyhole technique was used with short-term follow-up (6 months). A later publications from the same author [138], with a follow-up of 36 months, demonstrated that the rate of recurrence with this technique was high (37 %). In these three studies, the mesh used was a pure ePTFE mesh. This would lead to the conclusion that one should avoid the keyhole technique if this material is chosen. The Sugarbaker repair should be performed instead.

Reported series using other meshes with the keyhole technique (ePTFE-PP mesh) show a low recurrence rate (4.1 % and 3 %, respectively) [135, 137]. Reports of performing the keyhole technique with a pure ePTFE mesh had recurrence rates of 37, 56, and 72.7 % [133, 146, 133]. The only series that reported a large number of patients showed a low rate of recurrence with the sandwich technique [142].

In summary, the quality of evidence for the various surgical techniques for parastomal hernia repair is low and precludes firm conclusions. RCTs would be ideal to compare the various techniques of parastomal hernia repair, as none has been reported to date.

## Section 10: New technologic developments

### From robotic surgery to NOTES and single-port surgery: Is there currently any role in ventral hernia repair?

#### D. Lomanto

*Search terms:* Animal, Hernia, abdominal surgery/Ventral hernia, Umbilical, Incisional hernia, prosthesis implantation, Laparoscopy, Suture technique and Instrumentations, Swine, Endoscopy/methods, Endoscopy/trends, Endoscopy Gastrointestinal Methods, Surgical Procedures, Minimally invasive, Robot, Robotic Surgery, Robotic Device, Endoscopic Surgery, Laparoscopy.

*Searching machines*: PubMed, Embase, and Medline (2003–2011) were searched.

##### Statements

**Table:** 

Level 4	Robot-assisted ventral hernia is a feasible alternative to laparoscopic repair of ventral hernia
	Intracorporeal suturing under direct visualization allows stable suture fixation of the mesh
	Helicoid tackers and transabdominal sutures contribute to postoperative pain

##### Recommendations

**Table:** 

Grade C	More studies must be conducted on the feasibility, practicality, and success of robot-assisted ventral hernia repair

Laparoscopic surgery requires a high degree of special resolution, dexterity, and technical skills as a result of the lack of depth perception, tactile sensation, and force feedback. New technologies have been developed to improve the ergonomics and the drawbacks of minimally invasive surgical robotic devices. In any surgical procedure, and especially in laparoscopic surgery, technical skills, experience, decision making, and manual skills are major predictors of outcome. If a surgical manipulator computer-controlled device can improve performance and outcome, patients will benefit [159, 160], especially in a procedure where the learning curve is steep like hernia repair [161, 162]. Since the first successful laparoscopic repair in 1993 [163] and subsequently the advent of this surgical manipulator, many groups worldwide have tried to experience and the benefits of the use of robotic device in ventral hernia repair [164–166].

##### Comments

Few studies have been published that analyze the benefits of robotic devices in ventral hernia repair. More studies must be conducted on the feasibility, practicality, and success of the robot-assisted ventral hernia repair. Schluender et al. [163] showed that the robot-assisted laparoscopic repair of ventral hernia using intracorporeal suturing allowed for stable suture fixation under direct visualization and eliminated the need for tackers. Tayar et al. [164] confirmed the benefits of the da Vinci system for intracorporeal suturing in humans.

#### Natural orifice transluminal endoscopic surgery (NOTES)

*Search terms:* Animal, Colon, Hernia, Ventral, Incisional, Umbilical hernia, Prosthesis Implantation, Surgical Mesh, Surgical instrumentation, Swine, Endoscopy/methods, Endoscopy/trends, Endoscopy Gastrointestinal Methods, Surgical Procedures, Minimally invasive surgery, Natural Orifice Transluminal Endoscopic Surgery, Natural Orifice Surgery, Surgical Wound Infection/prevention, Intraperitoneal Infection, Laparoscopy.

*Searching machines*: PubMed, Embase, and Medline (2003–2011) were searched. The search identified 11 relevant papers: 10 with evidence level 4 and 1 with evidence level 1b.

##### Statements

**Table:** 

Level 1	Mesh placement via NOTES is technically feasible but has a high infection rate
Level 4	The risk of infection is much higher than in open or laparoscopic transabdominal ventral hernia repair
	The vaginal wall seems to be a safer entry site compared to the gastric wall

##### Recommendations

**Table:** 

Grade C	Access and development of an effective delivery device (which eliminates the contamination of the mesh through a colonized route) is necessary before trials can be started in humans
	Comparative studies are necessary to verify the feasibility and success rate of this new methodology

Surgery and especially endolaparoscopic surgery has gone through a fast-paced revolution in the last two decades. Flexible endoscopy has been refined with additional features like narrow banding imaging and high definition; the wide clinical use of robotic devices like Zeus and da Vinci; and the development of new and combined energy sealing devices like Ultracision (Ethicon Endosurgey, USA), Ligasure (Covidien, USA), and recently Thunderbeat (Olympus, Japan). These innovations and the use of more information technology—like wireless technology are completely changing the way surgery will be performed in the near future. In 2004, a new concept of natural orifice transluminal endoscopic surgery (NOTES) started fascinating surgeons, scientists, and industries worldwide. The innovative concept of performing surgery inside the abdominal cavity by accessing it through a natural orifice (mouth, vagina, or other) represent another potential innovation [167–171].

The actual benefits of NOTES, however, have yet to be proven because most research into this exciting new field is focused on small trials involving animal models [172]. Although substantial knowledge has been gained from these studies in a relatively short time, many safety issues have to be considered especially when challenging the time-honored basic surgical principles of the avoidance of unnecessary enterotomies by going beyond the natural borders of the mucosa [173]. Some human experience has been gained, but the technique is currently considered experimental; it has received much criticism and skepticism amid the enthusiasm [172]. A review of human NOTES experiences shows that so far, all have been performed under the guidance, assistance, or monitoring of concomitant laparoscopy in a hybrid setting. Multiple constraints in performance of NOTES have been identified [176]. Principally, present endoscopic systems are not designed with sufficient dexterity for NOTES procedures. Performing NOTES with today’s endoscopic instrumentation is technically difficult as a result of the limited endoscopic field of visualization and considerable constraints in the ability to maneuver the instruments within the small confines of the peritoneal cavity. In NOTES, off-axis operation is often necessary. Tasks such as tissue approximation and dissection require independent coordination of two instruments approaching from different angles. However, the parallelism of standard endoscopic fixtures limits the degree of freedom for optimal surgical maneuvers and does not permit much triangulation of endoscopically deployed instruments to approach the surgical target. For these reasons, experimental NOTES in humans have thus far focused on technically less challenging procedures. Hypothetical benefits of NOTES include the following: the entire abdominal fascia at risk for herniation can be visualized; the chance of port-site hernias is reduced; the cosmetic result is better because of minimal or no scarring; and there is less pain [177].

##### Comments

The platform and technology necessary to perform NOTES are still under development. Most of the reported surgical procedures are hybrid procedures. Comparisons should look at both simple and difficult procedures. Delivery of a foreign body (mesh) through a colonized natural orifice may increase chronic mesh infection compared to laparoscopic techniques. Results from studies drew different conclusions. Few studies reported an increased mesh infection rate in their subjects [178–180], while others [181–183] showed that bacterial contamination and intra-abdominal morbidities were not encountered during surgeries when using the transvaginal approach compared to the transgastric route. Ventral hernia repair using the NOTES approach seems to be safe and feasible in both experimental groups and in the few initial reports in humans [177–179, 182–186].

#### Single-port surgery

*Search terms:* Hernia, Ventral, Umbilical, Incisional Hernia, Laparoscopy/methods Surgical Instruments, SILS, Single port, Surgical Mesh, SPA, Single Port Access, Surgical Mesh, laparoscopic surgery, minimally invasive surgery.

*Searching machines*: PubMed, Embase, and Medline databases (2005–2011) and resulted in five relevant articles (level 4).

##### Statements

**Table:** 

Level 4	Single-port access ventral hernia repair appears to be safe for experienced endolaparoscopic surgeons. It may decrease parietal trauma and scarring in patients prone to incisional hernia and may be associated with a decrease in the rate of port-site hernia compared to multiport laparoscopy

##### Recommendations

**Table:** 

Grade C	Single-port access ventral hernia repair seems to be a safe and feasible alternative option to conventional laparoscopy in selected cases, but further RCTs are needed

In the last few years, minimally invasive surgery has continued to develop by further reducing surgical injury and scars. This new approach (NOTES) has created a lot of enthusiasm, but several issues and challenges have arisen that need to be resolved before full clinical acceptance [187–189]. While improving on these procedures, the idea of reducing the number and size of ports, so-called single incision access surgery evolved. Through a small incision (1.5–2.5 cm), the single-port device can be inserted, which can then allow access of multiple sites for the laparoscope and instruments to carry out the surgery. Early reports of different procedures have been published. It appears that the cosmetic advantage offered by single-port endolaparoscopic surgery makes this approach an attractive option for patients who desire an additional benefit of cosmesis. Further clinical studies involving large series of patients are needed to confirm the benefits and advantages of single-port endolaparoscopic surgery over standard procedures. There have been a few case reports published on inguinal [190, 191] and ventral hernia repair, with promising results [192–196].

##### Comments

The literature reviewed demonstrates that the procedure is feasible, safe, and reproducible. No intraoperative complications were observed. Standard instruments were used. Patients were discharged on the first day after surgery.

## Section 11: Lumbar and other unusual hernias

### Are lumbar and other unusual hernias suitable for laparoscopic repair?

#### K. A. LeBlanc, R. H. Fortelny

*Search terms:* Flank hernia repair, flank hernia repair with mesh, lumbar hernia repair, lumbar hernia repair with mesh, unusual hernias of the abdominal wall, spigelian hernia, spigelian hernia repair, lateral incisional hernia, traumatic lumbar hernia, Grynfelt OR Grynfelt’s hernia, Petit OR Petit’s hernia; the above AND repair, the above AND laparoscopy, lumbar hernia AND lumbar muscles AND paralysis, lumbar hernia AND lumbar muscles AND paralysis AND bulge, lumbar hernia AND lumbar muscles AND paralysis AND nephrectomy, lumbar hernia AND nephrectomy.

*Searching machines*: PubMed, Embase, and Medline (2000–2011) were searched.

##### Statements: Lumbar hernia

**Table:** 

Level 2b	Laparoscopic repair of lumbar hernia (with mesh) is superior to open repair with mesh in terms of morbidity but not recurrence rate
Level 4	There does not appear to be any distinct advantage of any method of repair for the “standard” fascial defect of lumbar hernias

##### Recommendations: Lumbar hernia

**Table:** 

Grade B	Options for repair of lumbar hernias include open repair with or without mesh in any position, and laparoscopic repair with mesh in any position. However, the laparoscopic repair is preferred because of reduced postoperative morbidity

##### Statements: Spigelian hernia

**Table:** 

Level 2b	Laparoscopic repair is superior because of reduced morbidity rates and length of hospital stay
Level 4	The placement of mesh is preferred either by the laparoscopic or the open method

##### Recommendations: Spigelian hernia

**Table:** 

Grade B	The use of mesh to repair these hernias by both approaches is recommended. However, the laparoscopic repair is preferred because of lower postoperative morbidity and reduced length of hospital stay. This represents an “upgraded” recommendation because of the clear superiority of the use of mesh for these hernias

##### Introduction

These two types of hernias are rare. Although most surgeons will have an opportunity to repair a spigelian hernia within their careers, many will never see a true lumbar hernia throughout their working career as a result of its extreme rarity, although its incidence may be increasing because of the more frequent use of the lumbar approach for anterior fusion of the lumbar spine. However, many of these bulges are the result of intercostal nerve injury and subsequent paralysis of the flat muscles of the abdominal wall.

The first suggestion of the existence of the lumbar hernias was by Barbette in 1672, but the first publication regarding these entities was by Garangeot in 1731. It is believed that the first surgical repair of a strangulated lumbar hernia occurred in 1750 by Ravaton. However, Petit and Grynfeltt’s names are associated with these hernias rather than the other surgeons because they provided the first anatomic description of the inferior lumbar space (Petit in 1783) and the superior lumbar space (Grynfeltt in 1866). The boundaries of the inferior lumbar hernia are the latissimus dorsi muscle posteriorly, the external oblique muscle anteriorly, and the iliac crest inferiorly. The boundaries of the superior lumbar hernia are the 12th rib superiorly, the internal oblique muscle anteriorly, and the erector spinae muscle posteriorly.

Selby described traumatic acquired lumbar hernia in 1906, and Kelton noted incisional acquired lumbar hernia in 1939. In 1951, Kretchmer published the first study of 11 of these latter hernias after renal surgery [197]. The ratio of congenital and acquired hernias has remained stable over time, with 80 % in the latter category. The etiology of the acquired defects has changed, however. Infectious etiology has declined from 17 to 2 %, whereas incisional hernias have increased from 10 to 31 % [198]. The laparoscopic approach to the repair of the lumbar hernia was first described by Burick and Parascandola [199] in 1996. Currently there are many methods and meshes to repair all of these defects.

Similar to the lumbar hernias, the name of the spigelian hernia is credited to someone who clarified the anatomic description of the entity, Adriaan van den Spieghel (1578–1625). This hernia occurs at the level of the semicircular line where the fascias of the oblique and transversus muscles begin to split to for the two separate layers of the abdominal musculature. Generally the overlying external oblique fascia remains intact, making this herniation interstitial and more difficult to diagnose. These entities are more common than that of the lumbar hernias.

##### Discussion

In this account, we have dealt with lumbar and spigelian hernias separately because they are separate entities. We searched the PubMed and Embase databases as well as the Cochrane register using the search terms noted above for publications that appeared from 1960 to 2011. Not unexpectedly, few publications could be used for an evidence-based systematic review on the treatment of both types of hernias. The relevant publications consisted of case series that included at least five cases. We excluded single case reports.

We were also charged to investigate the unusual hernias that were located in other locations. With these we were able to identify 48 articles but all were either single case series or did not really deal with the repair of the hernia. Hence none could be used for the systematic review. The search culled 35 articles under “flank hernia,” but these were either case reports or did not address any aspect of hernioplasty. No articles were found that dealt with lateral bulging after a denervation injury after nephrectomy or anterior lumbar disc surgery. Seventy-nine publications were found that described lumbar hernias or their repair. Fourteen were case series of fewer than five cases. Two were solely anatomic descriptive articles, and one was a publication that duplicated already published data. We were able to include in the review 12 papers, which contained five or more patients and one prospective randomized study. No publication had level of evidence 1a, 1b, 2a, 2c, or 3.

Moreno-Egea et al. [200] reported on a prospective nonrandomized study of 16 patients, 15 of whom were postnephrectomy and one after trauma. Mesh was used in all of the repairs, with seven done by the open method and nine by a laparoscopic approach. They found that the open repair was associated with a longer operative time, a longer length of stay, higher morbidity, and more recurrences. There were no recurrences in the laparoscopic group compared to three in the open group (*p* = 0.4). They concluded that the laparoscopic repair was “more efficient and profitable.” This level 2b evidence supports laparoscopic repair.

Twelve articles provided evidence at level 4. Of these, six were performed with the open technique only [201–206]. Four were performed solely laparoscopically [207–210]. One paper included patients who were treated with both open or laparoscopic method [198]. From these reports, a total of 123 patients could be evaluated. In four patients, the method of repair could not be determined from the article. The methods of repair used in the other 119 patients are shown in Table 3.

**Table 3:** Lumbar repair—method and number of patients

Method	Sutured repair	Preperitoneal mesh	Onlay mesh	Intraperitoneal mesh	Mesh location not stated
Open	28	17	11	6	8
Laparoscopic	0	0	0	32	17

Unfortunately, only 108 patients listed in Table 3 had adequate follow-up. These consisted of 28 patients with an open sutured repair, 31 with an open repair with mesh in any location, and 49 patients with a laparoscopic repair with mesh in any location. No recurrences were reported in any group of patients, but the length of follow-up varied from 1 to 40 months for the entire patient population. Given these results, it would appear that any method of repair for the lumbar hernia—sutured or with mesh placed by any method or location—appears to be an acceptable operation.

Bathla et al. [211] performed a review of the literature and included two cases of their own. On the basis of this review and their experience, their conclusion was that a combined open and laparoscopic repair using transfascial sutures with or without bone anchors was the best method to treat these difficult hernias. Stumpf et al. [212] performed cadaver dissections to address the problem and concluded that mesh should be used and placed in the sublay position between the internal and external oblique muscles.

The spigelian search revealed 397 articles. Of these, spigelian hernia was noted in 391, but only 95 of these reported on repair of these defects with a sufficient number of patients. The “spigelian hernia repair AND adult” search identified 263 articles, which included 95 case reports. Ninety non-English-language papers were excluded. Forty-five articles dealt with the radiologic assessment or diagnosis alone. Sixteen publications were not relevant to the review and were excluded, thus leaving 16 articles, each of which included five or more patients and provided details of the repair performed. Additionally, one publication was identified from another database. From all these 17 papers, no usable data could be found at levels of evidence 1a, 1b, 2a, 2c, 3, or 5.

The search identified only one prospective randomized trial of open versus laparoscopic repair of the spigelian hernia [213]. In this small RCT, patients were randomized to either an open or laparoscopic repair arm, with 11 patients in each arm. All meshes were placed in the preperitoneal space except for three in the laparoscopic group, where the mesh was placed in the intraperitoneal space. The laparoscopic repair was accompanied by lower postoperative morbidity (*p* < 0.05) and reduced length of hospital stay (*p* < 0.001). The authors concluded that the laparoscopic extraperitoneal repair should be the preferred treatment for these hernias.

The majority of the level 4 evidence articles were series of patients with an open sutured repair. Several were identified that included the diagnosis and treatment of the hernia but could not be included because no morbidity or follow-up data were provided. Length of follow-up varied greatly among the series. The cumulative data are shown in Table 4. It is obvious that the use of mesh is preferred. In the three series that included patients who underwent repair without the use of a mesh, the recurrence rate was 4–14 %. There were no recurrences in any series that included mesh in the repair either with the open or laparoscopic technique. The mesh was placed in the intraperitoneal, extraperitoneal, or intra-aponeurotic locations without the development of a recurrence.

**Table 4:** Summary of spigelian hernia data

Study	No. of repairs	Open sutured	Open mesh	Laparoscopic mesh	Recurrence rate
Artioukh [214]	19	19			0
Campanelli [215]	32		32		0
Celdrán [216]	9		9		0
Larson [217]	81	75	5	1	3/75 (4 %), no mesh
Malazgirt [218]	34		34		0
Mittal [219]	10			10	0
Moreno-Egea [220]	28 (17 open but not stated if mesh was used)			11	0
Mouton [221]	35	21	14		3/21 (14 %), no mesh
Palanivelu [107]	8			8	0
Patie [222]	6			6	0
Saber [223]	8			8	0
Sanchez-Montes [224]	6		6		0
Singer [225]	8	8			0
Vos [226]	25	20	5		1/20 (5 %), no mesh
Weiss [227]	9	9			0
Total	318	152	105	44	7/318 (2.2 %)
Recurrence rate	7/318 (2.2 %)	7/152 (4.6 %)	0	0	

## Section 12: Education

### Education and training in laparoscopic ventral hernia repair

#### D. Lomanto

*Search terms:* Hernia/abdominal surgery/Ventral hernia, Umbilical, Incisional hernia, Learning curve, Education/Laparoscopy, General surgery/education, Surgical procedures/operative education, Surgical procedures/operative psychology, Teaching/methods, Internship/residency, Competency based education, Computer assisted instruction.

*Searching machines*: PubMed, Embase, and Medline (2000–2011) were searched.

##### Statements

**Table:** 

Level 1	A structured laparoscopic training program in hernia repair improves operator proficiency in the operating room
Level 2c	Specialist centers seem to perform better than general surgical units, especially for endoscopic repairs
Level 4	There is a positive correlation between LVHR simulator training and performance in the operating room
	Operative performance can be greatly affected by surgical judgment and intraoperative decision making
	Surgeons with advanced laparoscopic skills are more likely to perform LVHR. Most with limited experience will begin after working with a preceptor
	The Global Operative Assessment of Laparoscopic Skills–Incisional Hernia (GOALS-IH) is easy to use, valid, and reliable for assessment of simulated LVHR
	A 1-day course may affect a surgeon’s practice
	It appears that the performance of 20 LVHR surgeons experienced in laparoscopic surgery leads to a plateau in recurrence rates and intraoperative complications

##### Recommendations

**Table:** 

Grade A	In departments performing incisional/ventral hernia repair, a structured laparoscopic training program should be introduced
Grade B	Complex hernia repairs should be done in specialized centers
Grade C	Laparoscopic training by virtual reality simulators may be done
	An added focus on decision-making skills in LVHR significantly affects operative performance
	Advanced laparoscopic skills should be acquired before mastery of LVHR
	Around 20 cases should be done to reach a plateau in performance of LVHR
	More studies must be conducted on the learning curve and on the best approach to integrate training in LVHR
Grade D	All surgeons graduating as general surgeons should acquire a profound knowledge of the commonly performed surgical repairs for conventional abdominal wall hernia repair by the onlay, sublay, and inlay methods
	Hernia repair under supervision of about 15 to 20 cases is ideal and necessary before a surgeon should work independently
	A structured laparoscopic hernia training program might improve surgical outcomes
	Complex abdominal wall hernia surgery (multiple recurrences, chronic pain, mesh infection) should be performed by a hernia specialist

Medical education is undergoing a paradigm shift from the traditional experience-based model to a program that requires documentation of proficiency [228].

Laparoscopic surgery requires a high degree of special resolution, dexterity, and technical skills. An initial training period is usually required for the majority of surgeons to become proficient in complex procedures by continuous repetition of these tasks [229–233]. Clinical outcome and complication rates are dependent on operator experience in those procedures. Surgeons who are less experienced in laparoscopic surgery and in LVHR will have higher complication rates. These results will be demonstrated by smaller scars, less postoperative pain, shorter hospital stay, lower recurrence rates, fewer infectious complications compared to open repair, and lower overall cost.

Surgeons recognize technical issues, operative decision making, and manual skills as major predictors of outcome [160, 234]. A learning curve for a specific procedure can be evaluated by means of operative times, but mainly the rate of conversions (for endolaparoscopic surgery) and complications. In the case of hernia repair, it is generally accepted that the learning curve for performing endoscopic inguinal hernia repair is longer than for open Lichtenstein repair, although the Lichtenstein technique also has a learning curve with respect to prevention of recurrence and chronic groin pain. However, this learning curve seems to be shorter than that for the endoscopic techniques [160, 161]. This is especially the case for endoscopic TEP repair as a result of a limited working space and different appreciation of the usual anatomical landmarks seen from inside the peritoneal cavity or through an anterior approach. There appears to be a higher rate of rare but serious complications with laparoscopic repair, especially during the learning curve period. Adequate patient selection and training might minimize these risks of infrequent but serious complications in the learning curve period [235–239].

Similarly for ventral hernia, the surgical treatment has undergone a paradigm shift in terms of repair, from simple suture repair to mesh repair to the first successful laparoscopic repair in 1991 [163]. LVHR, like any other minimally invasive procedure, offers advantages but has its own challenges: the challenge of any other minimally invasive procedure, familiarity of new instruments (meshes, tackers, suture passers, etc.), and familiarity of laparoscopic anatomy (though minimal for an experienced laparoscopic surgeon) [163, 240–242]. The exact definition of the learning curve in laparoscopic procedures is unclear [234]. Possible factors that may influence the learning curve may include the surgeon’s experience with other laparoscopic procedures and instrumentation, knowledge of laparoscopic anatomy, standardization of surgical technique, and reduction of operative time and complication rate. On the basis of limited or no data on training or on the learning curve of ventral hernia repair, we suggest that a minimal training of 15–20 cases is required by experienced laparoscopic surgeons to tackle the difficulties of the technique and to achieve comparable clinical outcome in terms of complications, operating time, and recurrence [162, 243–244]. Supervision by an experienced surgeon may help reduce the learning curve, as suggested in several studies for other procedures, including inguinal hernia repair [231, 238, 239].

Complex abdominal wall hernia repair should be performed in specialized centers. These centers seem to have better outcomes than general surgical units, especially for endoscopic repairs and complex inguinal hernia surgery (multiple recurrences, chronic pain, mesh infection, etc.), and such hernias should best be treated by a hernia specialist [230, 247, 248]. It is unclear whether subspecialty training, center volume, and/or surgeon volume are equally important to determine the outcome [245], but for many procedures, the observed associations between hospital volume and operative mortality are largely mediated by surgeon volume [246].

A structured laparoscopic training program in hernia repair improves operator outcomes in the operating room and surgical outcome because this allows the surgeon to learn directly from experts about the challenges encountered during the procedures and how to overcome them. This, followed by supervision and/or proctoring, can be useful in achieving good clinical results and to shorten the learning curve. Even a 1-day course may affect the surgeon’s practice, especially regarding hernia repair [247, 248].

In the era of information technology and computer simulation, training in ventral hernia has been positively influenced by these new devices [249–251]. Laparoscopic training by virtual reality simulators has shown a proven benefit in terms of improved operator performance in the operating room, even in LVHR [252–254].

##### Comments

Only few studies have been performed to analyze the learning curve. Time spent learning is needed to decrease the incidence of conversion and intraoperative complications in LVHR.

Although the laparoscopic technique of ventral hernia is conceptually straightforward, adhesiolysis requires more advanced skills.

## Questions for the future

### M. Smietanski, K. LeBlanc

These guidelines provide an answer to many of the questions concerning laparoscopic treatment of ventral hernias. However, many questions remain unanswered. Most surgeons agree that the material used for abdominal wall reinforcement should be individualized for specific groups of patients. However, the exact methods to enable such a choice for the individual patients are unclear. What is known is that with the assistance of the surgical community, the development of meshes that are engineered to meet the needs of our patients will continue to progress. The establishment of specifications for industry concerning the properties of meshes (prosthesis, scaffolds) has become the biggest challenge for scientists in relation to more effective repair of hernias. In the last year, the first models of the anterior abdominal wall have been created, but they describe only the average type of human body. The influence of BMI, age, and gender on the anterior abdominal wall movements should improve the understanding the forces acting on the prosthesis used to repair the abdominal wall. Such data should be complemented by studies designed to improve our knowledge of the histological differences in musculofascial architecture and its changes with various human body types.

Experimental studies are needed to understand mesh–fixation–abdominal wall system behavior, including the following:

1. Modeling of anterior abdominal wall in different groups of patients (e.g., BMI, gender, age).
2. Histopathological and mechanical description of the properties of the fascia in different patients.
3. Modeling of mesh–fascia fixation behavior for different systems.
4. Long-term in vivo studies to understand the changes in prosthesis properties caused by tissue ingrowth.
5. Studies on polymers and weave structure to develop different meshes for individual use.
6. Better clarification of the biological causes of herniation and whether these can be genetically linked.

In addition, surgeons have to understand that the prosthesis is not the main reason for a successful hernia repair. Properties of the mesh—porosity, elasticity, and the architecture of the weave—will be designed to complete the properties of prosthesis-fixation-abdominal wall system and should be understood as a part of this system. Different meshes and fixation devices express different properties at various time points after the operation, so the fixation algorithm will differ with the various materials. It is important to consider the fact that less fixation can lead to recurrence, but the use of too many tackers or sutures can increase postoperative pain.

It also seems that we have to widen the scope of scientific experiments to understand the changes in the prosthesis properties caused by tissue ingrowth and scar remodeling in long-term observation. Such studies together with clinical trials could answer the question on permanent or absorbable fixation. The ingrowth of the mesh and tissue also reveals the issue of abdominal wall preparation before the mesh will be placed and fixed (the so-called landing zone).

All of the above factors will aid in the future design of individual meshes for different hernia locations, its dimensions, fascial structure combined with anterior abdominal wall imaging (3D computed tomographic or magnetic resonance imaging models) and perhaps biological products used for mesh construction. We will perhaps realize the development of 3D printing and the use of this equipment for creation of “personal” mesh. The future exploration of our field should encompass the identification of the specific mesh types and methods to implant them on the basis of the clinical comorbidities of the patient who is being treated. For mesh repairs, we need to define the appropriate size of pores as well as determine the strongest product with the least risk of infection while providing a very low rate of recurrence; these await identification.

Clinical questions, based on our ongoing observations of outcomes, can only be answered if more clinical studies are performed:

1. Are there any differences in clinical outcome depending on the meshes used? (More studies are needed to assess the value of different materials, but also their safety.)
2. Do the different mesh/fixation systems influence the short- and long-term clinical outcome?
3. Is the construction of the mesh the cause of prolonged postoperative pain symptoms? If so, how can postoperative pain be prevented?
4. Are the commonly used prostheses really safe, and do they lessen adhesion formation?
5. How can we avoid local complications such as adhesions to the meshes or tackers or inflammation in the peritoneal cavity?
6. What is the precise indication for the use of laparoscopic ventral hernia surgery?
7. Should laparoscopic ventral hernia surgery be individualized?
8. Will the concept of functional restoration limit the use of laparoscopic methods in the future even if the risk of complications is proven to be higher?
9. What role will biomaterials play in LVHR in the future?
10. What conclusions can be drawn from large databases (e.g., EuraHS, HerniaMed, AHS Collaboration), and how could it help surgeons in the proper choice of the material and approach to repair a hernia?
11. Are hybrid procedures (open with the assistance of the laparoscopic approach) a better alternative for complex hernia repair, such as difficult lumbar hernias?
12. Is a new direction of mesh implant development shifting to the introduction of products that reduce or eliminate pain while providing a long-lasting repair within our grasp?
13. Will genetic engineering allow us to avoid and/or treat defects in collagen synthesis to avoid or repair hernias with native tissues?
14. Can the mesh materials be designed so that antibiotics can be infused into them to prevent infection at implantation or treat infection after implantation?

## Electronic supplementary material

Below is the link to the electronic supplementary material.

Supplementary material 1 (DOCX 66 kb)

