# Supplementary material for: Guidelines for laparoscopic treatment of ventral and incisional abdominal wall hernias (International Endohernia Society [IEHS])—Part III
Source: Surg Endosc. 2013 Sep 17;28(2):380–404. doi: 10.1007/s00464-013-3172-4 (PMC3936126; doi:10.1007/s00464-013-3172-4)
Supplement: Supplementary file 1 — Supplementary material 1 (DOCX 66 kb) [file 464_2013_3172_MOESM1_ESM.docx]

**List of references**

1. Eriksen JR, Gögenur I, Rosenberg J (2008) Choice of mesh for laparoscopic ventral hernia repair.Hernia 11:481-492 **(level 4)**
2. Shankaran V, Weber DJ, Reed, RL, Luchette FA (2011) A Review of Available Prosthetics for Ventral Hernia Repair. Annals of Surgery 253( 1):16-26 **(level 2A)**
3. Halm JA, de Wall LL, Steyenberg MC, Jeekel J, Lange JF (2007) Intraperitoneal polypropylene mesh hernia repair complicates subsequente abdominal surgery.World J Surg Feb.; 31 (2): 423-429 **(level 4)**
4. Deeken CR, Faucher KM, Matthews BD (2012) A review of the composition, characteristics and effectiveness of barrier mesh protheses utilized for laparoscopic ventral hernia repair. Surg Endosc 26(2):566-75.**(level 4)**
5. Huber A, McCabe GP, Boruch AV, Medberry C, Honerlaw, M, Badylak SF (2012) Polypropylene-containing synthetic mesh devices in soft tissue repair: A meta-analysis.J Biomed Mater Res B Appl Biomater. 100(1):145-54**(level 1A)**
6. Moreno-Egea A, Carrillo-Alcaraz A, Soria-Aledo V (2013) Randomized clinical trial of laparoscopic hernia repair comparing titanium-coated lightweight mesh and medium-weight composite mesh. Surg Endosc 27(1):231-9. **(level 1B)**
7. Colon MJ, Telem DA, Chin E, Weber K, Divino CM, Nguyen SQ (2011) Polyester Composite Versus PTFE in Laparoscopic Ventral Hernia Repair. JSLS 15 (3):305-308 **( level 3)**
8. Chelala E, Debardemaeker Y, Elias B, Charara F, Dessily M, Allé JL (2010) Eighty-five redo surgeries after 733 laparscopic treatments for ventral and incisional hernia: adhesion and recurrence analysis.Hernia 14:123-129 **(level 4)**
9. Jenkins ED, Yom V, Melman L, Brunt LM, Eagon JC, Frisella MM, Matthews BD (2010) Prospective evaluation of adhesion characteristics to intraperitoneal mesh and adhesiolysis-related complications during laparoscopic re-exploration after prior ventral hernia repair. Surg Endosc 24:3002-3007 **(level 4)**
10. Wassenaar EB, Schoenmaeckers EJP, Raymakers JTFJ, Rakic S (2010) Subsequent abdominalsurgery after laparoscopic ventral and incisional hernia repair with an expanded

polytetra-fluoroethylene mesh: a single institution experience with 72 reoperations. Hernia 14:137-142 **(level 4)**

1. Heniford BT, Park A, Ramshaw BJ, Voeller G (2003) Laparoscopic Repair of Ventral Hernias.Nine Years Experience With 850 Consecutive Hernias. Annals of Surgery 238, (3), 391-400 **(level 4)**
2. Koehler RH, Begons D, Berger D, Carey S, LeBlanc K, Park A, Ramshaw B, Smoot R, Voeller G(2003) Minimal Adhesions to ePTFE Mesh After Laparoscopic Ventral Incisional Hernia Repair: Reoperative Findings in 65 Cases. Journal of the Society of Laparoscopic Surgeons 7 (4) 335-340 **(level 4)**
3. Berger D, Bientzle M (2009) Polyvinylidene fluoride: a suitable mesh material for laparoscopic

incisional and parastomale hernia repair! Hernia 13:167-172 **(level 4)**

1. Fortelny RH, Petter-Pucher AH, Glaser KS, Offner F, Benesch T, Rohr M (2010) Adverse effects ofpolyvinylidene fluoride-coated polypropylene mesh used for laparoscopic intraperitoneal onlay repairof incisional hernia. British Journal of Surgery 97:1140-1145 **(level 4)**
2. Chowbey PK, Sharma A, Khullar R, Mann V, Baijal M, Vashistha A (2000) Laparoscopic VentralHernia Repair. Journal of Laparoendoscopic & Advanced Surgical Techniques 10:79-84 **(level 4)**
3. Misra M (2012) personal communication **(level 5)**
4. Penttinen R, Grönroos JM (2008) Mesh repair of common abdominal hernias: a review onexperimental and clinical studies.Hernia 12:337-344 **(level 4)**
5. Conze J, Rosch R, Klinge U, Weiss C, Anurov M, Titkowa S, Oettinger A, Schumpelick V (2004)Polypropylene in the intra-abdominal position: Influence of pore size and surface area.Hernia 8:365-372 **(level 5)**
6. Borrazzo EC, Belmont MF, Boffa D, Fowler DL (2004) Effect of prosthetic material on adhesionformation after laparoscopic ventral hernia repair in a porcine model. Hernia 8:108-112 **(level 5)**
7. Jacob BP, Hogle NJ, Durak E, Kim T, Fowler DL (2007) Tissue ingrowth and bowel adhesionformation in an animal comparative study: polypropylene versus Proceed versus Parietex Composite. Surg Endosc 21:629-633 **(level 5)**
8. Schug-Paß C, Tamme C, Tannapfel A, Köckerling F 2006) A lightweight polypropylene mesh(TiMesh) for laparoscopic intraperitoneal repair of abdominal wall hernias. Surg Endosc 20: 402-409**(level 5)**
9. Duffy AJ, Hogle NJ, LaPerle KM, Fowler DL (2004) Comparison of two composite meshes usingtwo fixation devices in a procine laparoscopic ventral hernia repair model. Hernia 8:358-364 **(level 5)**
10. Zinther NB, Wara P, Friis-Andersen H (2010) Intraperitoneal onlay mesh: an experimental studyof adhesion formation in a sheep model. Hernia 14:283-289 **(level 5)**
11. Schug-Pass C, Sommerer F, Tannapfel A, Lippert H, Köckerling F (2009) The use of compositemeshes in laparoscopic repair of abdominal wall hernias: are there differences in biocompatibily? Surg Endosc 23:487-495 **(level 5)**
12. Deeken CR, Matthews BD (2012) Comparison of contracture, adhesion, tissue ingrowth, and histologic response characteristics of permanent and absorbable barrier meshes in a procine model of laparoscopic ventral hernia repair. Hernia Feb;16(1):69-76. **(level 5)**
13. Bellows CF, Smith A, Malsbury J, Helton WS.(2013) Repair of incisional hernia with biological prosthesis: a systematic review of current evidence The American Journal of Surgery Jan;205(1):85-101.**(level 2A)**
14. Cavasco M, Itani KMF.(2012) Ventral Hernia Repair with Synthetic, Composite, and Biologic Mesh: Characteristics, Indication, and Infection Profile. Surgical Infections 13: 209-215**(level 2A)**
15. Harth KC, Rosen MJ(2009) Major Complications Associated With Xenograft Biologic Mesh Implantation in Abdominal Wall Reconstruction. Surgical Innovation 16: 324-329**(level 2C)**
16. Miserez M, G. Grass, C. Weiss, H. Stützer, S. Sauerland, Neugebauer EAM.(2010) Closure of the LAPSIS trial. British Journal of Surgery 97, 1598**(level 1B)**
17. Cobb GA, Shaffer J.(2005) Cross-Linked Acellular Porcine Dermal Collagen Implant in Laparoscopic Ventral Hernia Repair: Case-Controlled Study of Operative Variables and Early Complications Int Surg, 90, 24-29**(level 3)**
18. Franklin Jr ME, Trevino JM, Portillo G, Vela I, Glass JL, Gonzalez JJ.(2008) The use of procine small intestinal submucosa as a prosthetic matieral for laparoscopic hernia repair in infected and potentially contaminated fields: long-term follow-up Surg Endosc 22, 19-41-1946**(level 4)**
19. UpToDate Jun 11, 2010. Reconstructive Materials used in surgery: Classification and host response. **(level 5)**
20. Usher, FC, Gannon JP (1959) A new plastic mesh for replacing tissue defects, experimental studies. Arch Surg 78:131**(level 5)**
21. Usher, FC, Ochsner J, Tuttle LLD Jr. (1958) Use of Marlex in incisional hernias Am Surg 24:969 **(5)**
22. Clave A, Yahi H, Hammou JC, Montanari S, Gounon P, Clave H (2010) Polypropylene as a reinforcement in pelvic surgery is not inert: comparative analysis of 100 explants. Int Urogynecol J 21:261-270 **(level 4)**
23. Klinge U, Klosterhalfen B, Muller M, Schumpelick V (1999) Foreign body reaction to meshes used for the repair of abdominal wall hernias Eur J Surg 165: 665-673 **(level 4)**
24. Klosterhalfen B, Junge K, Hermanns B, Klinge U (2002) Influence of implantation interval on the long-term biocompatibility of surgical mesh Br J Surgery 89(8): 1043-1048 **(level 4)**
25. Offner FA (2004) Pathophysiology and pathology of the foreign-body reaction to mesh implants. In: Schumpelick V, Nyhus LM, eds. *Meshes: Benefits and Risks*. New York, NY: Springer; (2004) 161-169 **(level 5)**
26. Costello CR, Bachman SL, Grant SA, Cleveland DS, Loy TS, Ramshaw BJ (2007) Characterization of heavyweight and lightweight polypropylene prosthetic mesh explants from a single patient Surg Innov 14(3) 168-176 **(level 4)**
27. Costello CR, Bachman SL, Ramshaw BJ, Grant SA (2007) Materials characterization of explanted polypropylene hernia meshes J of Biomedical Materials Research Part B: Applied Biomaterials DOI 10.1002/jbmb 44-49 **(level 4)**
28. Ratner B, Hoffman AS, Schoen FJ, Lemons JE (1996) Biomaterials Science. San Diego, CA: Academic Press 243-254 **(level 5)**
29. Coda A, Bendavid R, Botto-Micca F, Bossotti M, Bona A (2003) Structural alterations of prosthetic meshes in humans **(level 4)**
30. Bracco P, Brunella V, Trossarelli L, Coda A, Botto-Micca F (2005 Comparison of polypropylene and polyethylene terephthalate (Dacron) meshes for abdominal wall hernia repair: A chemical and morphological study Hernia 9: 51-55 **(level 4)**
31. Cozad MJ, Grant DA, Bachman SL, Grant DN, Ramshaw BJ, Grant SA (2010) Materials characterization of explanted polypropylene, polyethylene terephthalate, and expanded polytetrafluoroethylene composites: Spectral and thermal analysis. J of Biomedical Materials Research B: Applied Biomaterials 455-462 **(level 4)**
32. Anthony T, Bergen P, Kim L, Henderson M, Fahey T, Rege R, Turnage R (2000) Factors affecting recurrence following incisional herniorrhaphy. World journal of surgery 24**:**95-101(**Level 4)**
33. Manninen MJ, Lavonius M, Perhoniemi VJ (1991) Results of incisional hernia repair. A retrospective study of 172 unselected hernioplasties. The European journal of surgery = Acta chirurgica 157**:**29-31(**Level 4)**
34. van 't Riet M, Steyerberg EW, Nellensteyn J, Bonjer HJ, Jeekel J (2002) Meta-analysis of techniques for closure of midline abdominal incisions. The British journal of surgery 89**:**1350-1356(**Level 1a)**
35. Paul A, Korenkov M, Peters S, Kohler L, Fischer S, Troidl H (1998) Unacceptable results of the Mayo procedure for repair of abdominal incisional hernias. EUROPEAN JOURNAL OF SURGERY 164**:**361-367(**Level 4)**
36. Read R, Yoder G (1989) Recent trends in the management of incisional herniation. Archives of Surgery 124**:**485-488(**level 4)**
37. Flum DR, Horvath K, Koepsell T (2003) Have outcomes of incisional hernia repair improved with time? A population-based analysis. Annals of surgery 237**:**129-135(**Level 3)**
38. Pearl RK (1989) Parastomal hernias. World journal of surgery 13**:**569-572(**Level 4)**
39. Takagi H, Sugimoto M, Kato T, Matsuno Y, Umemoto T (2007) Postoperative incision hernia in patients with abdominal aortic aneurysm and aortoiliac occlusive disease: a systematic review. European journal of vascular and endovascular surgery : the official journal of the European Society for Vascular Surgery 33**:**177-181(**Level 3 )**
40. Hall KA, Peters B, Smyth SH, Warneke JA, Rappaport WD, Putnam CW, Hunter GC (1995) Abdominal wall hernias in patients with abdominal aortic aneurysmal versus aortoiliac occlusive disease. American journal of surgery 170**:**572-575; discussion 575-576(**Level 4)**
41. Adye B, Luna G (1998) Incidence of abdominal wall hernia in aortic surgery. American journal of surgery 175**:**400-402(**Level 4)**
42. Christou NV, Jarand J, Sylvestre JL, McLean AP (2004) Analysis of the incidence and risk factors for wound infections in open bariatric surgery. Obesity surgery 14**:**16-22(**Level 4)**
43. Arribas D, Elia M, Artigas C, Jimenez A, Aguilella V, Martinez M (2004) Incidence of incisional hernia following vertical banded gastroplasty. Hernia 8**:**135-137(**Level 4)**
44. Sugerman HJ, Kellum JM, Jr., Reines HD, DeMaria EJ, Newsome HH, Lowry JW (1996) Greater risk of incisional hernia with morbidly obese than steroid-dependent patients and low recurrence with prefascial polypropylene mesh. American journal of surgery 171**:**80-84(**Level 4)**
45. O'Hare JL, Ward J, Earnshaw JJ (2007) Late results of mesh wound closure after elective open aortic aneurysm repair. European journal of vascular and endovascular surgery : the official journal of the European Society for Vascular Surgery 33**:**412-413(**Level 4)**
46. Bevis PM, Windhaber RA, Lear PA, Poskitt KR, Earnshaw JJ, Mitchell DC (2010) Randomized clinical trial of mesh versus sutured wound closure after open abdominal aortic aneurysm surgery. The British journal of surgery 97**:**1497-1502(**Level 2a)**
47. Pans A, Elen P, Dewe W, Desaive C (1998) Long-term results of polyglactin mesh for the prevention of incisional hernias in obese patients. World journal of surgery 22**:**479-482; discussion 482-473(**Level 3)**
48. Strzelczyk J, Czupryniak L, Loba J, Wasiak J (2002) The use of polypropylene mesh in midline incision closure following gastric by-pass surgery reduces the risk of postoperative hernia. Langenbeck's archives of surgery / Deutsche Gesellschaft fur Chirurgie 387**:**294-297(**Level 4)**
49. Strzelczyk JM, Szymanski D, Nowicki ME, Wilczynski W, Gaszynski T, Czupryniak L (2006) Randomized clinical trial of postoperative hernia prophylaxis in open bariatric surgery. The British journal of surgery 93**:**1347-1350(**Level 2b)**
50. Gutierrez de la Pena C, Medina Achirica C, Dominguez-Adame E, Medina Diez J (2003) Primary closure of laparotomies with high risk of incisional hernia using prosthetic material: analysis of usefulness. Hernia 7**:**134-136(**Level 3)**
51. Llaguna OH, Avgerinos DV, Nagda P, Elfant D, Leitman IM, Goodman E (2011) Does prophylactic biologic mesh placement protect against the development of incisional hernia in high-risk patients? World journal of surgery 35**:**1651-1655(**Level 4)**
52. http://www.clinicaltrials.gov(2009)Primary_mesh_closure_of_abdominal_midline_wounds_(PRIMA)(NCT00761475).http://clinicaltrials.gov/ct2/show/record/NCT00761475?term=PRIMA&rank=3 (2009) (Expected **Level 1b)**
53. Carne PW, Robertson GM, Frizelle FA (2003) Parastomal hernia. The British journal of surgery 90**:**784-793(**Level 4)**
54. Rieger N, Moore J, Hewett P, Lee S, Stephens J (2004) Parastomal hernia repair. Colorectal disease : the official journal of the Association of Coloproctology of Great Britain and Ireland 6**:**203-205(**Level 4)**
55. Hansson BM, de Hingh IH, Bleichrodt RP (2007) Laparoscopic parastomal hernia repair is feasible and safe: early results of a prospective clinical study including 55 consecutive patients. Surgical endoscopy 21**:**989-993(**Level 3)**
56. Berger D, Bientzle M (2009) Polyvinylidene fluoride: a suitable mesh material for laparoscopic incisional and parastomal hernia repair! A prospective, observational study with 344 patients. Hernia 13**:**167-172(**Level 3)**
57. Bayer I, Kyzer S, Chaimoff C (1986) A new approach to primary strengthening of colostomy with Marlex mesh to prevent paracolostomy hernia. Surgery, Gynecology & Obstetrics 163**:**579-580(**Level 4)**
58. Janes A, Cengiz Y, Israelsson LA (2004) Preventing parastomal hernia with a prosthetic mesh. Archives of surgery (Chicago, Ill : 1960) 139**:**1356-1358(**Level 2a)**
59. Helgstrand F, Gogenur I, Rosenberg J (2008) Prevention of parastomal hernia by the placement of a mesh at the primary operation. Hernia 12**:**577-582(**Level 2a)**
60. Hammond TM, Huang A, Prosser K, Frye JN, Williams NS (2008) Parastomal hernia prevention using a novel collagen implant: a randomised controlled phase 1 study. Hernia 12**:**475-481(**Level 2b)**
61. Janes A, Cengiz Y, Israelsson LA (2009) Preventing parastomal hernia with a prosthetic mesh: a 5-year follow-up of a randomized study. World journal of surgery 33**:**118-121; discussion 122-113(**Level 3)**
62. Serra-Aracil X, Bombardo-Junca J, Moreno-Matias J, Darnell A, Mora-Lopez L, Alcantara-Moral M, Ayguavives-Garnica I, Navarro-Soto S (2009) Randomized, controlled, prospective trial of the use of a mesh to prevent parastomal hernia. Annals of surgery 249**:**583-587(**Level 2a)**
63. Shabbir J, Chaudhary BN, Dawson R (2012) A systematic review on the use of prophylactic mesh during primary stoma formation to prevent parastomal hernia formation. Colorectal disease : the official journal of the Association of Coloproctology of Great Britain and Ireland 14**:**931-936(**Level 1a)**
64. Tam KW, Wei PL, Kuo LJ, Wu CH (2010) Systematic review of the use of a mesh to prevent parastomal hernia. World journal of surgery 34**:**2723-2729(**Level 2A)**
65. Wijeyekoon SP, Gurusamy K, El-Gendy K, Chan CL (2010) Prevention of parastomal herniation with biologic/composite prosthetic mesh: a systematic review and meta-analysis of randomized controlled trials. Journal of the American College of Surgeons 211**:**637-645(**Level 1A)**
66. Wijeyekoon Sanjaya P, Gurusamy Kurinchi S, El-Gendy K, Chan Christopher LH, Williams Norman S (2010) Prosthetic mesh for prevention of parastomal herniation. Cochrane Database of Systematic Reviews, DOI: 10.1002/14651858.CD008905, (**Expected Level 1a)**
67. Seiler CM, Bruckner T, Diener MK, Papyan A, Golcher H, Seidlmayer C, Franck A, Kieser M, Buchler MW, Knaebel HP (2009) Interrupted or continuous slowly absorbable sutures for closure of primary elective midline abdominal incisions: a multicenter randomized trial (INSECT: ISRCTN24023541). Annals of surgery 249**:**576-582(**Level 2a)**
68. Brown Steven R, Tiernan J (2005) Transverse verses midline incisions for abdominal surgery. Cochrane Database of Systematic Reviews, DOI: 10.1002/14651858.CD005199.pub2, (**Level 1a)**
69. Halm JA, Lip H, Schmitz PI, Jeekel J (2009) Incisional hernia after upper abdominal surgery: a randomised controlled trial of midline versus transverse incision. Hernia 13**:**275-280(**Level 2b)**
70. Fassiadis N, Roidl M, Hennig M, South LM, Andrews SM (2005) Randomized clinical trial of vertical or transverse laparotomy for abdominal aortic aneurysm repair. The British journal of surgery 92**:**1208-1211(**Level 2b)**
71. Seiler CM, Deckert A, Diener MK, Knaebel HP, Weigand MA, Victor N, Buchler MW (2009) Midline versus transverse incision in major abdominal surgery: a randomized, double-blind equivalence trial (POVATI: ISRCTN60734227). Annals of surgery 249**:**913-920(**Level 1b)**
72. Rahbari NN, Knebel P, Diener MK, Seidlmayer C, Ridwelski K, Stoltzing H, Seiler CM (2009) Current practice of abdominal wall closure in elective surgery - Is there any consensus? BMC surgery 9**:**8(**Level 5)**
73. Hodgson NC, Malthaner RA, Ostbye T (2000) The search for an ideal method of abdominal fascial closure: a meta-analysis. Annals of surgery 231**:**436-442(**Level 1A)**
74. Weiland DE, Bay RC, Del Sordi S (1998) Choosing the best abdominal closure by meta-analysis. American journal of surgery 176**:**666-670(**Level 2A)**
75. Rucinski J, Margolis M, Panagopoulos G, Wise L (2001) Closure of the abdominal midline fascia: meta-analysis delineates the optimal technique. The American surgeon 67**:**421-426(**Level 2b)**
76. Gupta H, Srivastava A, Menon GR, Agrawal CS, Chumber S, Kumar S (2008) Comparison of interrupted versus continuous closure in abdominal wound repair: a meta-analysis of 23 trials. Asian journal of surgery / Asian Surgical Association 31**:**104-114(**Level 1b)**
77. Diener MK, Voss S, Jensen K, Buchler MW, Seiler CM (2010) Elective midline laparotomy closure: the INLINE systematic review and meta-analysis. Annals of surgery 251**:**843-856(**Level 1a)**
78. Rahbari NN, Knebel P, Kieser M, Bruckner T, Bartsch DK, Friess H, Mihaljevic AL, Stern J, Diener MK, Voss S, Rossion I, Buchler MW, Seiler CM (2012) Design and current status of CONTINT: continuous versus interrupted abdominal wall closure after emergency midline laparotomy - a randomized controlled multicenter trial [NCT00544583]. Trials 13**:**72(**Expected Level 1b)**
79. Israelsson LA, Jonsson T (1993) Suture length to wound length ratio and healing of midline laparotomy incisions. The British journal of surgery 80**:**1284-1286(**Level 4)**
80. Israelsson LA, Jonsson T (1996) Incisional hernia after midline laparotomy: a prospective study. The European journal of surgery = Acta chirurgica 162**:**125-129(**Level 3)**
81. Millbourn D, Cengiz Y, Israelsson LA (2011) Risk factors for wound complications in midline abdominal incisions related to the size of stitches. Hernia : the journal of hernias and abdominal wall surgery 15**:**261-266(**Level 2a)**
82. Gruppo M, Mazzalai F, Lorenzetti R, Piatto G, Toniato A, Ballotta E (2012) Midline abdominal wall incisional hernia after aortic reconstructive surgery: a prospective study. Surgery 151**:**882-888(**Level 3)**
83. Millbourn D, Israelsson LA (2004) Wound complications and stitch length. Hernia : the journal of hernias and abdominal wall surgery 8**:**39-41(**Level 3)**
84. Israelsson LA, Jonsson T (1997) Overweight and healing of midline incisions: the importance of suture technique. The European journal of surgery = Acta chirurgica 163**:**175-180(**Level 4)**
85. Cengiz Y, Blomquist P, Israelsson LA (2001) Small tissue bites and wound strength: an experimental study. Archives of surgery (Chicago, Ill : 1960) 136**:**272-275**(Level 5)**
86. Harlaar JJ, van Ramshorst GH, Nieuwenhuizen J, Ten Brinke JG, Hop WC, Kleinrensink GJ, Jeekel H, Lange JF (2009) Small stitches with small suture distances increase laparotomy closure strength. American journal of surgery 198**:**392-395 **(level 5)**
87. Millbourn D, Cengiz Y, Israelsson LA (2009) Effect of stitch length on wound complications after closure of midline incisions: a randomized controlled trial. Archives of surgery (Chicago, Ill : 1960) 144**:**1056-1059(**Level 2a)**
88. Harlaar JJ, Deerenberg EB, van Ramshorst GH, Lont HE, van der Borst EC, Schouten WR, Heisterkamp J, van Doorn HC, Cense HA, Berends F, Stockmann HB, Vrijland WW, Consten EC, Ottow RT, Go PM, Hermans JJ, Steyerberg EW, Lange JF (2011) A multicenter randomized controlled trial evaluating the effect of small stitches on the incidence of incisional hernia in midline incisions. BMC surgery 11**:**20(**Expected Level 1b)**
89. Sajid MS, Bokhari SA, Mallick AS, Cheek E, Baig MK (2009) Laparoscopic versus open repair of incisional/ventral hernia: a meta-analysis.Am J Surg 197(1):64-72. **(level 1A)**
90. Rives J, Pire JC, Flament JB, Convers G (1977) Treatment of large eventrations (apropos of 133 cases).Minerva Chir. 32(11):749-56. **(level 2C)**

# Gagner M, Milone L, Gumbs A, Turner P (2010) Laparoscopic repair of left lumbar hernia after laparoscopic left nephrectomy. JSLS 14(3):405-9. **(level 4)**

# Majeski J (2009) Open and laparoscopic repair of Spieghelian hernia. Int Surg 94(4):365-9. **(level 4)**

# Hilling DE, Koppert LB, Keijzer R, Stassen LP, Oei IH (2009) Laparoscopic correction of umbilical hernias using a transabdominal preperitoneal approach: results of a pilot study

Surg Endosc 23(8):1740-4. **(level 4)**

# Palanivelu C, Vijaykumar M, Jani KV, Rajan PS, Maheshkumaar GS, Rajapandian S (2006) Laparoscopic transabdominal preperitoneal repair of Spieghelian hernia.

JSLS 10(2):193-8. **(level 4)**

1. McKay R, Haupt D (2006) Laparoscopic repair of low abdominalwall hernias by tack fixation to the cooper ligament. Surg Laparosc Endosc Percutan Tech 16(2):86-90. **(level 4)**
2. Bhandarkar DS, Katara AN, Shah RS, Udwadia TE (2005) Transabdominalpreperitonealrepair of a port-site incisional hernia. J Laparoendosc Adv Surg Tech A 15(1):60-2. **(level 4)**

# Shekkariz B, Graziottin TM, Gholami S, Lu HF, Yamada H, Duh QY, Stoller ML (2001) Transperitoneal preperitoneal laparoscopic lumbar incisional herniorrhaphy. J Urol 166(4):1267-9. **(level 4)**

1. Miserez M, Penninckx F (2002) Endoscopic totally preperitonealventral herniarepair. Surg Endosc 16(8):1207-13. **(level 4)**

# Koksal N, Altinli E, Celik A, Oner I (2004) Extraperitoneal laparoscopic approach to Spieghelian hernia combined with groin hernias. Surg Laparosc Endosc Percutan Tech 14(4):204-6. **(level 4)**

# Tarnoff M, Rosen M, Brody F (2002) Planned totally extraperitoneal laparoscopic Spieghelian hernia repair. Surg Endosc 16(2):359**. (level 4)**

1. Ramirez O, Ruas E, Dellon A (1990) Components Separation Method for closure of abdominal wall defects: an anatomic and clinical study. Plast Recontsr Surg 86:526. **(level 3)**
2. Rosen MJ, Jin J, McGee MF, Williams C, Marks J, Ponsky JL (2007) Laparoscopic component separation in the single-stage treatment of infected abdominal wall prosthetic removal. Hernia, 11(5):435-40**.(level 3)**
3. Harth KC, Rosen MJ (2010) Endoscopic versus open component separation in complex abdominal wall reconstruction. Am J Surg 199(3):342-6**.(level 3)**
4. Harth KC, Rose J, Delaney CP, Blatnik JA, Halaweish I, Rosen MJ (2011) Open versus endoscopic component separation: a cost comparison. Surg Endosc 25(9):2865-70**.(level 3)**
5. Albright E, Diaz D, Davenport D, Roth JS (2011) The component separation technique for hernia repair: a comparison of open and endoscopic techniques. Am Surg 77(7):839-43**.(level 3)**
6. Giurgius M, Bendure L, Davenport DL, Roth JS. (2012) The endoscopic component separation technique for hernia repair results in reduced morbidity compared to the open component separation technique. Hernia 16(1):47-51**.(level 3)**
7. Bachman SL, Ramaswamy A, Ramshaw BJ. (2009) Early results of midline hernia repair using a minimally invasive component separation technique. Am Surg 75(7):572-7**.(level 3)**
8. Parker M, Bray JM, Pfluke JM, Asbun HJ, Smith CD, Bowers SP (2011)
   Preliminary experience and development of an algorithm for the optimal use of the laparoscopic component separation technique for myofascial advancement during ventral incisional hernia repair. J Laparoendosc Adv Surg Tech A 21(5):405-10**.(level 4)**
9. Losanoff JE, Richman BW, Jones JW (2002) Endoscopically assisted "componentseparation" method for abdominal wall reconstruction. J Am Coll Surg 195(2):288-291.(level 4)
10. Tadeo-Ruiz G, Picazo-Yeste J.S, Moreno-Sanz C, Herrero-Bogajo ML.(2010) Parastomal Hernias: Background, current status and future prospects. Cir Esp 87(6):339-49.**(Level 5)**
11. Hiranyakas A, Ho YH..(2010) Laparoscopic parastomal hernia repair. Dis Colon Rectum. 53(9):1334-6.**(Level 5)**
12. De Raet J, Delvaux G, Haentjens P, Van Nieuwenhave Y..(2008) Waist circunference is an independent risk factor for the development of a parastomal hernia after permanent colostomy. Dis Colon Rectum 51(12):1806-9.**(level 4)**
13. Pilgrim CH, McIntyre R, Bailey M. .(2010) Prospective audit of parastomal hernia: prevalence and associated comorbidities. Dis Colon Rectum 53(1):71-6.**(Level 2C)**
14. Rubin MS, Schoetz JR DJ, Mathews J.B..(1994) Parastomal hernia. Is stoma relocation superior to fascial repair?.Arch Surg 129:413-8.**(Level 3)**
15. Cheung MT,ChiaNH,Chiu WY. .(2001) Surgical treatment of parastomal hernia complicating sigmoid colostomies. Dis Colon Rectum 44:266-70.**(Level 3)**
16. Tekkis PP, Kocher HM, Payne JG. .(1998) The continuing challenge of parastomal hernia: failure of a novel polypropylene mesh repair. Ann R CollSurgEngl 80:184-7.**(Level 5)**
17. White TJ, Santos MC, Thompson JS. .(1998) Factors affecting wound complications in repair of ventral hernias. Am Surg 64:276-80.**(Level 3)**
18. Pastor DM, Pauli EM, Koltun WA, Haluck RS, Shope TR, Poritz LS. .(2009)Parastomal hernia repair: a single center experience. JSLS. 13(2):170-5.**(Level 3b)**
19. McLemore EC, Harold KL, Efron JE, Laxa BU, Young-Fadok TM, Heppell JP..(2007) Parastomal hernia: short-term outcome after laparoscopic and conventional repairs. SurgInnov. 14(3):199-204. **(Level 3b)**
20. Muysoms EE, Hauters PJ, Van Nieuwenhove Y, Huten N, Claeys DA..(2008)Laparoscopic repair of parastomal hernias: a multi-centre retrospective review and shift in technique. ActaChir Belg. 108(4):400-4. **(Level 3b)**
21. Mizrahi H, Bhattacharya P, Parker MC. .(2012) Laparoscopic slit mesh repair of parastomal hernia using a designated mesh:long-term results. SurgEndosc 26(1):267-70. **(Level 4)**
22. Liu F, Li J, Wang S, Yao S, Zhu Y. .(2011) Effectiveness analysis of laparoscopic repair of parastomal hernia using CK Parastomal patch. ZhongguoXiu Fu Chong JianWaiKeZaZhi. 25(6):681-4.**(Level 4)**
23. Jani K. .(2010) Laparoscopic paracolostomy hernia repair: a retrospective case series at atertiary care center. SurgLaparoscEndoscPercutan Tech. 20(6):395-8.**(Level 4)**
24. Wara P, Andersen LM..(2011) Long-term follow-up of laparoscopic repair of parastomal hernia using a bilayer mesh with a slit. SurgEndosc. 25(2):526-30.**(Level 4)**
25. Hansson BM, Bleichrodt RP, de Hingh IH. .(2010)Laparoscopic parastomal hernia repair using a keyhole technique results in a high recurrence rate. SurgEndosc. 2009;23(7):1456-9. **(Level 4)**
26. Zacharakis E, Hettige R, Purkayastha S, Aggarwal R, Athanasiou T, Darzi A, Ziprin P. .(2008) Laparoscopic parastomal hernia repair: a description of the technique and initialresults. SurgInnov. 15(2):85-9. **(Level 4)**
27. Saber AA, Rao AJ, Rao CA, Elgamal MH.(2008) Simplified laparoscopic parastomal hernia repair: the scroll technique.Am J Surg. 196(3):e16-8.**(Level 4)**
28. Craft RO, Huguet KL, McLemore EC, Harold KL..(2008) Laparoscopic parastomal hernia repair.Hernia. 12(2):137-40.**(Level 4)**
29. Berger D, Bientzle M. .(2007) Laparoscopic repair of parastomal hernias: a single surgeon's experience in 66 patients. Dis Colon Rectum. 50(10):1668-73.**(Level 4)**
30. Inan I, Gervaz P, Hagen M, Morel P. .(2007) Multimedia article. Laparoscopic repair of parastomal hernia using a porcine dermal collagen (Permacol) implant.Dis Colon Rectum. 50(9):1465.**(Level 4)**
31. Mancini GJ, McClusky DA 3rd, Khaitan L, Goldenberg EA, Heniford BT, Novitsky YW, Park AE, Kavic S, LeBlanc KA, Elieson MJ, Voeller GR, Ramshaw BJ. .(2007) Laparoscopic parastomal hernia repair using a nonslit mesh technique. SurgEndosc. 21(9):1487-91. **(Level 4)**
32. LeBlanc KA, Bellanger DE, Whitaker JM, Hausmann MG. .(2005)Laparoscopic parastomal hernia repair.Hernia. 9(2):140-4. **(Level 4)**
33. Safadi B..(2004) Laparoscopic repair of parastomal hernias: early results. SurgEndosc. 18(4):676-80. **(Level 4)**
34. Kozlowski PM, Wang PC, Winfield HN..(2001) Laparoscopic repair of incisional and parastomal hernias after major genitourinary or abdominal surgery. J Endourol. 15(2):175-9.**(Level 4)**
35. Voitk A. .(2000) Simple technique for laparoscopic paracolostomy hernia repair. Dis Colon Rectum. 43(10):1451-3.**(Level 4)**
36. Dumanian G. .(2002) Laparoscopic repair of paraostomy hernias. J Am Coll Surg. 195(2):293.**(Level 4)**
37. Wara P.(2011) Parastomal hernia repair. An update. Minerva Chir. 66(2):123-8.**(Level 5)**
38. Berger D. (2010)[Laparoscopic repair of parastomal hernia]. Chirurg. 81(11):988-92.**(Level 5)**
39. Nagy A, Jánó Z. (2010)[Parastomal hernias].MagySeb. 63(5):335-9.**(Level 5)**
40. Israelsson LA. (2010)[Parastomal hernia treatment with prosthetic mesh repair].Chirurg. 81(3):216-21. **(Level 5)**
41. Israelsson LA.(2008) Parastomal hernias. SurgClin North Am. 88(1):113-25, ix.**(Level 5)**
42. Voeller GR. (2007)Innovations in ventral hernia repair. SurgTechnol Int. 16:117-22.**(Level 5)**
43. Sugarbaker PH. (1985)Peritoneal approach to prosthetic mesh repair of parastomal hernias. Ann Surg 201:344-46.**(Level 4)**
44. Sauerland S, Walgenbach M, Habermalz B, Seiler CM, Miserez M. (2011)Laparoscopic versus open surgical techniques for ventral or incisional hernia repair. Cochrane Database Syst Rev. 16;(3):CD007781. **(Level 1A)**
45. Kapischke M, Schulz T, Schipper T, Tensfeldt J, Caliebe A. (2008)Open versus laparoscopic incisional hernia repair: something different from a meta-analysis. Surg Endosc. 22(10):2251-60. **(Level 1A)**
46. Ramsay CR et al. (2001)Statistical assessment of the learning curves of health technologies. Health Technol Assess 5: 1-79**. (Level4)**
47. Simons MP, Aufenacker T, Bay-Nielsen M, Bouillot JL, Campanelli G, Conze J, de Lange D, Fortelny R, Heikkinen T, Kingsnorth A, Kukleta J, Morales-Conde S, Nordin P, Schumpelick V, Smedberg S, Smietanski M, Weber G, Miserez M. (2009) European Hernia Society guidelines on the treatment of inguinal hernia in adult patients. Hernia 13: 343-403. **(Level 2A)**
48. Voitk AJ. (1998)The learning curve in laparoscopic inguinal hernia repair for the community general surgeon. Can J Surg 41: 446-50 **(Level3)**
49. Bencini L, Sánchez LJ(2004): Learning curve for laparoscopic ventral hernia repair. Am J Surg 187; 378-382 **(Level3)**
50. LeBlanc KA, Booth WV. (1993) Laparoscopic repair of incisional abdominal hernias using expanded polytetrafluoroethylene: preliminary findings. Surg Laparosc Endosc. 3:39–41**.(Level3)**
51. Ballantyne GH, Hourmont K, Wasielewski A. (2003) Telerobotic laparoscopic repair of incisional ventral hernias using intraperitoneal prosthetic mesh. JSLS. 7(1):7-14. PubMed PMID: 12722992 **(Level 3)**
52. Schluender S, Conrad J, Divino CM, Gurland B. (2003)Robot-assisted laparoscopic repair of ventral hernia with intracorporeal suturing. Surg Endosc. 17(9):1391-5. **(Level3)**
53. Tayar C, Karoui M, Cherqui D, Fagniez PL. (2007)Robot-assisted laparoscopic mesh repair of incisional hernias with exclusive intracorporeal suturing: a pilot study. Surg Endosc. 21(10):1786-9. **(Level3)**
54. Kalloo AN, Singh VK, Jagannath SB, Niiyama H, Hill SL, Vaughn CA, Magee CA, Kantsevoy SV (2004). Flexible transgastric peritoneoscopy: a novel approach to diagnostic and therapeutic interventions in the peritoneal cavity. Gastrointest Endosc 60:114–117. **(level 5)**
55. McGee M, Rosen M, Marks J,Onders RP, Chak A, Faulx A, Chen VK, Ponsky J.(2006) A primer on Natural Orifice Transluminal Endoscopic Surgery: building a new paradigm. Surg Innov 13-2: 86-93. **(level 4)**
56. Merrifield B, Wagh M, Thompson C. (2006) Peroral transgastric organ resection: a feasibility study in pigs. Gastrointest Endosc 63:693-97. **(level 5)**
57. Kantsevoy SV, Hu B, Jagannath CA,Vaughn CA, Beitler DM, Chung SS, Cotton PB, Gostout CJ, Hawes RH, Pasricha PJ, Magee CA, Pipitone LJ, Talamini MA, Kalloo AN.(2006) Transgastric endoscopic splenectomy – Is it Possible? Surg Endosc 20: 522-525. **(level5)**
58. Wagh M, Merrifield B, Thompson C. (2006) Survival studies after endoscopic transgastric oophorectomy and tubectomy in a porcine model. Gastrointest Endosc 63:473-78. **(level 5)**
59. Rattner D, Hawes R. (2006)Notes: Gathering Momentum. Gastroint Endosc 63:838-839**(Level 5)**
60. Hochberger J, Lamade W. (2005) Transgastric surgery in the the abdomen: the dawn of a new era? Gastroint Endosc 62:293-296. **(level 5)**
61. Jagannath SB, Kantsevoy SV, Vaughn CA,Chung SS, Cotton PB, Gostout CJ, Hawes RH, Pasricha PJ, Scorpio DG, Magee CA, Pipitone LJ, Kalloo AN. (2005) Peroral transgastric endoscopic ligation of fallopian tubes with long-term survival in a porcine model. Gastrointest Endosc 61:449-53.**(level 5)**
62. Pai R, Fong D, Bundga M. (2006)Transcolonic endoscopic cholecystectomy: a NOTES survival study in a porcine model. Gastroint Endoscopy 64:428-434. **(level 5)**
63. ASGE/SAGES Working Group on Natural Orifice Translumenal Endoscopic Surgery White Paper October 2005. ASGE; SAGES. Gastrointest Endosc. 2006 Feb;63(2):199-203. **(level 4)**
64. Powell B, Whang SH, Bachman SL, Astudillo JA, Sporn E, Miedema BW, Thaler K. (2010)Transvaginal repair of a large chronic porcine ventral hernia with synthetic mesh using NOTES. JSLS. 14(2):234-9. **(level 5)**
65. Earle DB, Desilets DJ, Romanelli JR. (2010)NOTES transgastric abdominal wall hernia repair in a porcine model. Hernia. 14(5):517-22. **(level 5)**
66. Jacobsen GR, Thompson K, Spivack A, Fischer L, Wong B, Cullen J, Bosia J, Whitcomb E, Lucas E, Talamini M, Horgan S. (2010) Initial experience with transvaginal incisional hernia repair. Hernia. 14(1):89-91. **(level 4)**
67. Buck L, Michalek J, Van Sickle K, Schwesinger W, Bingener J. (2008)Can gastric irrigation prevent infection during NOTES mesh placement? J Gastrointest Surg. 12(11):2010-4. **(level 5)**
68. Lomanto D, Chua HC, Myat MM, So J, Shabbir A, Ho L. (2009) Microbiological contamination during transgastric and transvaginal endoscopic techniques. J Laparoendosc Adv Surg Tech A. 19(4):465-9. **(level 5)**
69. Miedema BW, Bachman SL, Sporn E, Astudillo JA, Thaler K. (2009)Transgastric placement of biologic mesh to the anterior abdominal wall. Surg Endosc. 23(6):1212-8. Epub 2009 Mar. **(level 5)**
70. Lomanto D, Dhir U, So JB, Cheah WK, Moe MA, Ho KY. (2009)Total transvaginal endoscopic abdominal wall hernia repair: a NOTES survival study. Hernia. 13(4):415-9. **(level 5)**
71. Sporn E, Astudillo JA, Bachman SL, Mayfield TP, Thaler K, Miedema BW. (2009)Transgastric biologic mesh delivery and abdominal wall hernia repair in a porcine model. Endoscopy. 41(12):1062-8. **(level 5)**
72. Fong DG, Ryou M, Pai RD, Tavakkolizadeh A, Rattner DW, Thompson CC. (2007)Transcolonic ventral wall hernia mesh fixation in a porcine model. Endoscopy. 39(10):865-9.**(level 5)**
73. Fortelny RH, Petter-Puchner AH, Gruber-Blum S, Mika K, Brand J, Keibl C,Glaser KS, Redl H. (2011)The Feasibility of FS Mesh Fixation by a Transgastric Approach-An Important Benefit in Future NOTES Procedures? J Surg Res 171(1):80-6.**(level 5)**
74. Romanelli JR, Earle DB. (2009) Single-port laparoscopic surgery: an overview. Surg Endosc. 23:1419-27.**(level 5)**
75. Allemann P, Schafer M, Demartines N.(2010) Critical appraisal of single port access cholecystectomy. Br J Surg. 97:1476-80.**(level 2C)**
76. Tracy CR, Raman JD, Cadeddu JA, Rane A. (2008) Laparoendoscopic single-site surgery in urology: where have we been and where are we heading? Nat Clin Pract Urol. 5:561-8.**(level 5)**
77. Goo TT,Goel R, Lawenko M, Lomanto D.(2010)Laparoscopic transabdominal preperitoneal (TAPP) hernia repair via a single port. Surg Laparosc Endosc Percutan Tech. 20:389-90.**(level 3)**
78. Jacob BP, Tong W, Reiner M, Vine A, Katz LB. (2009)Single incision total extraperitoneal (one SITE) laparoscopic inguinal hernia repair using a single access port device. Hernia. 13:571-2.**(level 3)**
79. Bower CE, Love KM. (2011)Single incision laparoscopic ventral hernia repair. JSLS 15(2):165-8. **(level 3)**
80. Tsivian A, Tsivian M, Sidi AA.(2011) Laparoendoscopic Single-site Repair of Incisional Hernias after Urological Surgery. Urology. 78(3):715-8. **(level 3)**
81. Bucher P, Pugin F, Morel P. (2011)Single-port access prosthetic repair for primary and incisional ventral hernia: toward less parietal trauma. Surg Endosc. 25(6):1921-5. **(level 3)**
82. Roberts KE, Panait L, Duffy AJ, Bell RL. (2010)Single-port laparoscopic umbilical hernia repair. Surg Innov. 17(3):256-60, **(level 3)**
83. Podolsky ER, Mouhlas A, Wu AS, Poor AE, Curcillo PG 2nd.(2010) Single Port Access (SPA) laparoscopic ventral hernia repair: initial report of 30 cases. Surg Endosc. 24(7):1557-61**.(level 3)**
84. Kretchmer HL. (1951)Hernia of the Kidney. J Urol. 65:944-949 **(Level 5)**
85. Moreno-Egea A, Baena EG, Calle MC, Martınez JAT, Albasini JLA.(2007)Controversies in the Current Management of Lumbar Hernia. Arch Surg. 142:82-88**(Level2A)**
86. Burick AJ, Parascandola SA.(1996) Laparoscopic repair of a traumatic lumbar hernia: a case report. J LaparoendoscSurg 6:259–262**(Level 5)**

Moreno-Egea A,Torralba-Martinez JA,Morales G,Fernández T,Girela E,Aguayo-Albasini JL. (2005) Open vs laparoscopic repair of secondary lumbar hernias.SurgEndosc 19: 184–187. **(Level 2B)**

1. Carbonell AM, Kercher KW, Sigmon L, Matthews BD, Sing RF, J. S. Kneisl JS,Heniford BT.(2005) A novel technique of lumbar hernia repair using bone anchor fixation.Hernia 9: 22–26. **(Level 4)**
2. Cavallaro G, Sadighi A, Micelli M, Burza A, Carbone G, Cavallaro A.(2007) Primary Lumbar Hernia Repair: The open approach. EurSurg Res 39:88-92. **(Level 4)**
3. Hafner CD, Wylie JH, Brush BE. .(1963) Petit’s lumbar hernia: repair with Marlex mesh. Arch Surg 86:180-186**.(Level 4)**
4. Light HG. .(1983) Hernia of the inferior lumbar space. Arch Surg 118:1077-1080**.(Level 4)**
5. Alfredo Moreno-Egea, MD, Monica Mengual-Ballester, MD, Mar ́ıa Jose ́ Cases-Baldo ́, MD, and Jose ́ Luis Aguayo-Albasini, MD,.(2010) Repair of complex incisional hernias using double prosthetic repair: Single-surgeon experience with 50 cases. Surgery.148(1):140-4. **(Level 4)**
6. X. Zhou, J. O. Nve, G. Chen .(2004) Lumbar hernia: Clinical analysis of 11 cases. Hernia 8: 260–263**.(Level 4)**
7. Arca MJ, Heniford BT, Pokorny R, Wilson MA, Mayes J, Gagner M. .(1998) Laparoscopic repair of lumbar hernias. JACS 187(2):147-152. **(Level 4)**
8. Palanivelu C, Rangarajan M, John SJ, Madankumar MV, Senthilkumar K..(2008) Laparoscopic transperitoneal repair of lumbar incisional hernias: a combined suture and 'double-mesh' technique. Hernia.12(1):27-31**.(Level 4)**
9. Yavuz N, Ersoy YE, Demirkesen O, Tortum OB, Erguney S (2009) Laparoscopic incisional lumbar hernia repair. Hernia 13:281–286. **(Level 4)**
10. Tobias-Machado M, Rincon FJ, Lasmar MT, Zambon JP, Juliano RV, Wroclawski ER. .(2005) Laparoscopic surgery for treatment of incisional lumbar hernia.IntBraz J Urol 31(4):309-314. **(Level 4)**

Bathla L, Davies E, Fitzgibbons RJ Jr, Cemaj S.(2011) Timing of traumatic lumbar hernia repair: is delayed repair safe? Report of two cases and review of the literature. Hernia 15(2):205-9**.(Level 5)**

.Stumpf M, Conze J, Prescher A, Junge K, Krones CJ, Klinge U, Schumpelick V.(2009) The lateral incisional hernia: anatomical considerations for a standardized retromuscularsublay repair Hernia 13:293–297**.(Level 4)**

Moreno-Egea A, Carrasco L, Girela E, Mart ́ın JG, Aguayo JL, Canteras M..(2002) Open vs Laparoscopic Repair of Spieghelian Hernia. A Prospective Randomized Trial. Arch Surg.  137:1266-1268. **(Level 2B)**

1. Artioukh DY, Walker SJ. (1996) Spieghelianherniae: presentation, diagnosis and treatment. JRCollSurgEdinb 41:241-243**.(Level 4)**
2. Campanelli G, Pettinari D, Nicolosi FM, ContessiniAvesani E..(2005) Spieghelian Hernia. Hernia 9:3-5. **(Level 4)**
3. Celdrán A, Señaris J, Mañas J, Frieyro O. .(2007) The open mesh repair of Spieghelian hernia. Am J Surg 193:111-113**.(Level 4)**
4. Larson DW, Farley DR..(2002) Spieghelian Hernias: Repair and Outcome for 81 patients. World J Surg 26:1277-1281**. (Level 4)**
5. Malazgirt Z, Topgul K, Sokmen S, ErsinS,Turkcapar AG, Gok H, Gonullu N, Paksoy M, Ertem M..(2006) Spieghelian hernias: a prospective analysis of baseline parameters and surgical outcome of 34 consecutive patients. Hernia 10:326-330**.(Level 4)**
6. Mittal T, Kumar V, Khullar R, Sharma A, Soni V, Baijal M, Chowbey PK. .(2008) Diagnosis and management of Spieghelian hernia: A review of literature and our experience. J Minim Access Surg 4(4):95-98**.(Level 4)**
7. Moreno-Egea, Flores B, Girela E, Martin JG, Aguayo JL, Canteras M..(2002) Spieghelian hernia: bibliographical study and presentation of a series of 28 patients. Hernia 6:167-170**.(Level 4)**
8. Mouton WG, Otten KT, Weiss D, Naef M, Wagner HE..(2006) Preperitoneal mesh repair in Spieghelian hernia.IntSurg 91(5):262-264.
9. Patie NM, Tantia O, Sasmal PK, Khanna S, Sen B. .(2010) Laparoscopic Repair of Spieghelian Hernia: Our Experience. JSLS 20(2):2010.
10. Saber AA, Elgamal MH, Rao AJ, Osmer RL, Itawi EA. .(2008) Laparoscopic Spieghelian Hernia Repair: The Scroll Technique. Am Surg 74(2):108-112.
11. Sanchez-Montes I, Deysine M..(1998) Spieghelian hernias: a new repair technique using preshaped polypropylene umbrella plugs. Arch Surg 133(6):670-672.
12. Singer JA, Mansberger AR. .(1973) Spieghelian hernia. Arch Surg 107(4):515-517.
13. Vos DI, Scheltinga MRM. .(2004) Incidence and outcome of surgical repair of Spighelian hernia. Br J Surg 91:640-644.
14. Weiss Y, Lernau OZ, Nissan S. .(1974) Spieghelian Hernia. Ann Surg 180(6):836-83
15. Reznick RK, MacRae H: .(2007) Teaching surgical skills – changes in the wind. NEJM 2006, 21;355 (25)2664-9. **(5)**
16. DeTurris SV, Cacchione RN, Mungara A, Pecoraro A, Ferzli GS. .(2002) Laparoscopic herniorrhaphy: beyond the learning curve. J Am Coll Surg 194: 65-73 **(3).**
17. Edwards CC, Bailey RW. .(2000) Laparoscopic hernia repair: the learning curve. Surg Laparosc Endosc Percutan Tech 10: 149-53 **(3).**
18. Fallon WF, Jr., Wears RL, Tepas JJ, III. .(1993) Resident supervision in the operating room: does this impact on outcome? J Trauma 35: 556-60 **(3).**
19. Feliu-Pala X, Martin-Gomez M, Morales-Conde S, Fernandez-Sallent E. .(2001) The impact of the surgeon's experience on the results of laparoscopic hernia repair. Surg Endosc 15: 1467-70 **(level 3).**
20. Miserez M, Arregui M, Bisgaard T, Huyghe M, Van Bruwaene S, Peeters E, Penninckx F. .(2009) A standardized resident training program in endoscopic surgery in general and in laparoscopic totally extraperitoneal (TEP) inguinal hernia repair in particular. Surg Laparosc Endosc Percutan Tech 19: e125-e129 **(Level3).**
21. Ramsay CR, Grant AM, Wallace SA, Garthwaite PH, Monk AF, Russell IT.(2001) Statistical assessment of the learning curves of health technologies. Health Technol Assess 5: 1-79. **(Level3)..**
22. Haidenberg J, Kendrick ML, Meile T, Farley DR. .(2003) Totally extraperitoneal (TEP) approach for inguinal hernia: the favorable learning curve for trainees. Curr Surg 60: 65-8. **(Level3).**
23. Lau H, Patil NG, Yuen WK, Lee F. .(2002) Learning curve for unilateral endoscopic totally extraperitoneal (TEP) inguinal hernioplasty. Surg Endosc 16: 1724-8. **(Level3).**
24. Liem MS,van Steensel CJ, Boelhouwer RU, Weidema WF, Clevers GJ, Meijer WS, Vente JP, de Vries LS, van Vroonhoven TJ.(1996) The learning curve for totally extraperitoneal laparoscopic inguinal hernia repair. Am J Surg 171: 281-5**. (Level3).**
25. Neumayer LA,Gawande AA, Wang J, Giobbie-Hurder A, Itani KM, Fitzgibbons RJ Jr, Reda D, Jonasson O.(2005) Proficiency of surgeons in inguinal hernia repair: effect of experience and age. Ann Surg 242: 344-8. **(Level3).**
26. Robson AJ, Wallace CG, Sharma AK, Nixon SJ, Paterson-Brown S. .(2004) Effects of training and supervision on recurrence rate after inguinal hernia repair. Br J Surg 91: 774-7**. (Level3).**
27. Lomanto D, Iyer S, Shabbir A, Cheah WK: .(2006) Laparoscopic versus open ventral hernia repair: a prospective study. Surg Endosc 20(7);1030-5 **(Level2B).**
28. Heniford BT, Matthews BD, Box EA, Backus CL, Kercher KW, Greene FL, Sing RF.(2002) Optimal teaching environment for laparoscopic ventral herniorrhaphy. Hernia 6 (1) :17-20 **(Level3).**
29. Le Blanc K: .(2004) Laparoscopic Incisional and Ventral Hernia: Complications – How to avoid and handle it. Hernia 8:323-31 **(Level3).**
30. Salameh JR,Sweeney JF, Graviss EA, Essien FA, Williams MD, Awad S, Itani KM, Fisher WE. .(2002) Laparoscopic ventral hernia repair during the learning curve. Hernia 6(1):182-187 **(Level3).**
31. Arwa AL-Harazi, Vipan K, Simran S, Iwena W, Cheah WK, Lomanto D: .(2011) Laparoscopic Ventral Hernia Reapir: there is a learning curve. Proceedings Asia Pacific Hernia Society Congress, Hua Hin, Thailand Abstract book pp. 21. **(Level3).**
32. Halm EA, Lee C, Chassin MR. .(2002) Is volume related to outcome in health care? A systematic review and methodologic critique of the literature. Ann Intern Med 137: 511-20 **(Level1A).**
33. Birkmeyer JD,Stukel TA, Siewers AE, Goodney PP, Wennberg DE, Lucas FL.(2003) Surgeon volume and operative mortality in the United States. N Engl J Med 349: 2117-27. **(Level2C).**
34. Zerey M, Kercher KW, Sing RF, Ramshaw BJ, Voeller G, Park A, Heniford BT: .(2007)  Does a one-day course influence surgeon adoption of laparoscopic ventral herniorrhaphy? Surg Res. 138(2):205-8. **(Level3)**
35. Hwang J,Telem DA, Nguyen SQ, Chin EH, Weber KJ, Divino CM..(2010) A novel laparoscopic ventral herniorrhaphy training system Surg Laparosc Endosc Percutan Tech 20 (1): 16-18. **(Level3)**
36. Pugh C, Plachta S, Auyang E, Pryor A, Hungness E: .(2010) Outcome measures for surgical simulators: is the focus on technical skills the best approach? Surgery. 147(5):646-54 **(Level3)**
37. Seagull FJ,George I, Ghaderi I, Vaillancourt M, Park A.: .(2009) Surgical Abdominal Wall (SAW): A Novel Simulator for Training in Ventral Hernia Repair. Surg Innov. 16(4):330-6.**(Level3)**
38. Jain M, Tantia O, Khanna S,Sen B, Sasmal PK..(2009) Hernia endotrainer: results of training on self-designed hernia trainer box. J Lap Adv Surg Tech 19(4) 535-540**(Level3)**
39. Vaillancourt M, Ghaderi I, Kaneva P ,Vassiliou M, Kolozsvari N, George I, Sutton FE, Seagull FJ, Park AE, Fried GM, Feldman LS.(2011) LS GOALS-incisional hernia: a valid assessment of simulated laparoscopic incisional hernia repair. Surg Innov. 18(1):48-54. **(Level3)**
40. Ghaderi I, Vaillancourt M, Sroka G, Kaneva PA, Seagull FJ, George I, Sutton E, Park AE, Vassiliou MC, Fried GM, Feldman LS..(2011) Performance of simulated laparoscopic incisional hernia repair correlates with operating room performance. Am J Surg. 201(1):40-5**(Level2B)**
41. Pugh CM, DaRosa DA, Santacaterina S, Clark RE: .(2010) Faculty evaluation of simulation-based modules for assessment of intraoperative decision making. Surgery 149:4; 534-542. **(Level 3)**
